# Association between cord blood metabolites in tryptophan pathway and childhood risk of autism spectrum disorder and attention-deficit hyperactivity disorder

**DOI:** 10.1038/s41398-022-01992-0

**Published:** 2022-07-09

**Authors:** Ramkripa Raghavan, Neha S. Anand, Guoying Wang, Xiumei Hong, Colleen Pearson, Barry Zuckerman, Hehuang Xie, Xiaobin Wang

**Affiliations:** 1 Center on Early Life Origins of Disease, Department of Population, Family and Reproductive Health, Johns Hopkins University Bloomberg School of Public Health, Baltimore, MD USA; 2 Department of Pediatrics, Boston University School of Medicine and Boston Medical Center, Boston, MA USA; 3 Department of Biomedical Sciences & Pathobiology, Fralin Life Sciences Institute at Virginia Technology, Blacksburg, VA USA; 4 Department of Pediatrics, Johns Hopkins University School of Medicine, Baltimore, MD USA

**Keywords:** Autism spectrum disorders, ADHD

## Abstract

Alterations in tryptophan and serotonin have been implicated in various mental disorders; but studies are limited on child neurodevelopmental disabilities such as autism spectrum disorder (ASD) and attention-deficit hyperactivity disorder (ADHD). This prospective cohort study examined the associations between levels of tryptophan and select metabolites (5-methoxytryptophol (5-MTX), 5-hydroxytryptophan (5-HTP), serotonin, N-acetyltrytophan) in cord plasma (collected at birth) and physician-diagnosed ASD, ADHD and other developmental disabilities (DD) in childhood. The study sample (*n* = 996) derived from the Boston Birth Cohort, which included 326 neurotypical children, 87 ASD, 269 ADHD, and 314 other DD children (mutually exclusive). These participants were enrolled at birth and followed-up prospectively (from October 1, 1998 to June 30, 2018) at the Boston Medical Center. Higher levels of cord 5-MTX was associated with a lower risk of ASD (aOR: 0.56, 95% CI: 0.41, 0.77) and ADHD (aOR: 0.79, 95% CI: 0.65, 0.96) per Z-score increase, after adjusting for potential confounders. Similarly, children with cord 5-MTX ≥ 25th percentile (vs. <25th percentile) had a reduction in ASD (aOR: 0.27, 95% CI: 0.14, 0.49) and ADHD risks (aOR: 0.45, 95% CI: 0.29, 0.70). In contrast, higher levels of cord tryptophan, 5-HTP and N-acetyltryptophan were associated with higher risk of ADHD, with aOR: 1.25, 95% CI: 1.03, 1.51; aOR: 1.32, 95% CI: 1.08, 1.61; and aOR: 1.27, 95% CI: 1.05, 1.53, respectively, but not with ASD and other DD. Cord serotonin was not associated with ASD, ADHD, and other DD. Most findings remained statistically significant in the sensitivity and subgroup analyses.

## Introduction

Autism spectrum disorder (ASD) is characterized by social and communication disabilities as well as restrictive and repetitive behaviors and interests [1] with 1 in 54 children in the US diagnosed with ASD [2]. Attention-deficit hyperactivity disorder (ADHD), another neurodevelopmental disorder, is characterized by a persistent pattern of inattention, hyperactivity-impulsivity, or both, with a prevalence of about 8.8% in the U.S [3]. The etiology of ASD and ADHD is unknown, although both disorders have genetic as well as environmental underpinnings, including prenatal and perinatal factors [4]. A well-observed phenomenon is that both of these conditions often co-occur and are known to share symptoms [5]. However, it is unclear whether ASD and ADHD share a molecular pathway.

**Fig. 1:**
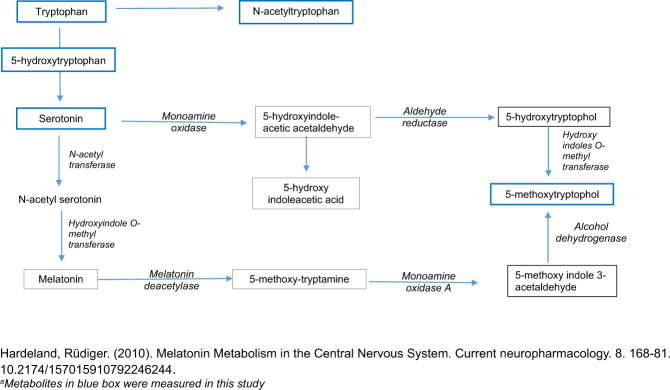
Pathway of tryptophan and its metabolites.

Tryptophan, an essential amino acid, serves as a precursor to neurotransmitters through multiple metabolic pathways (Fig. 1). Some of these are well known such as serotonin, 5-hydroxytryptophan (5-HTP), and melatonin [6] and have been implicated in a number of conditions including depression, mood disorders, ASD, and ADHD [7–13].

Beyond tryptophan, serotonin, and melatonin, very few studies to date have assessed other less known tryptophan metabolites, especially in the context of neurodevelopmental conditions. One such metabolite is 5-methoxytryptophol (5-MTX) [9] which is shown to exhibit diurnal rhythm in humans [14] and animal models [15–19]. Emerging evidence from clinical trials and other studies suggests that 5-MTX may be involved in major depressive disorder (MDD) [20, 21], developmental processes in humans [22, 23] and possesses cortical electrical activity [24–26]. While studies have hypothesized 5-MTX’s potential role in ASD [27, 28], to the best of our knowledge, none of the studies have formally examined this association in a prospective birth cohort, especially along with other metabolites in the tryptophan pathway.

Built on the previous observations and to address the major knowledge gaps, we set out to assess the association of tryptophan and select metabolites (serotonin, 5-MTX, 5-HTP, N-acetyltryptophan) measured in the cord blood plasma collected at birth and subsequent risk of physician diagnosis of ASD and ADHD. We will leverage a well-defined prospective birth cohort, which allows us to examine the temporal and dose-response associations of cord tryptophan metabolites and the risk of ASD, ADHD, and other developmental disabilities (DD), considering the extent of comorbidity between these conditions possibly suggesting a common underlying etiology [29].

## Methods

Detailed information on the participation and enrollment in the Boston Birth Cohort (BBC) has been discussed previously [30, 31]. Briefly, BBC is a preterm enriched cohort such that for every preterm (defined as <37 weeks of gestation) and/or low birth weight baby (defined as <2500 g), approximately two term and normal birth weight babies and their mothers were enrolled in the study. Shortly after delivery, mothers were approached to participate in the study and ~90% of whom consented [31]. Participants and non-participants did not differ on characteristics such as infant birth weight, maternal race/ethnicity, or other socio-demographic characteristics. Trained research staff used a standardized questionnaire to interview the mothers 24 to 72 h after birth. Pertinent clinical information, including laboratory reports, pregnancy complications, labor, and delivery course, and birth outcomes, was obtained by reviewing maternal and infant records.

A sub-set of the children originally enrolled continued to receive pediatric care at the Boston Medical Center (BMC), were followed up from birth to 21 years of age and were included in this study. These mother-infant dyads recruited at birth remained in the follow-up study from October 1, 1998 to June 30, 2018. As reported earlier, there were no major differences in the baseline demographic and clinical characteristics of those that continued to be part of postnatal follow-up and those that did not [32]. Of the total of 3165 that were followed up in the BBC, 996 subjects had sufficient cord plasma samples for metabolite analyses and met the definitions for ASD, ADHD, other DDs, or neurotypical development (eFig. 1).

Written informed consent was obtained from the mothers and depending on the age of the child, verbal or written consent was also obtained from the participating children. The database for research did not contain personal identifier information and is accessible only to authorized investigators. The Institutional Review Boards of Boston University Medical Center and the Johns Hopkins Bloomberg School of Public Health approved the study protocol. This study followed the Strengthening the Reporting of Observational Studies in Epidemiology (STROBE) reporting guideline for cohort studies.

### Exposure

Umbilical cord blood samples were collected at the time of delivery and were processed and fractioned into cells and plasma shortly after collection by the field team at BMC. Quantitative profiling of analytes including tryptophan and its select metabolites (serotonin, 5-MTX, 5-HTP, N-acetyltryptophan) from cord was assessed in a random sub-set of children as part of a metabolome panel and was measured using a liquid chromatography tandem mass spectrometry technique at the Broad Institute Metabolite Profiling Laboratory at the Massachusetts Institute of Technology. The distribution of most of the cord analytes was skewed and hence inverse normal transformation was used to render the distribution approximately normal. Details on laboratory methods, quality control, and data processing have been described elsewhere [33, 34].

### Outcome

For every postnatal clinical visit since 2003, we obtained primary and secondary diagnoses using *International Classification of Diseases, Ninth Revision (ICD-9)* or *International Classification of Diseases, Tenth Revision (ICD-10) codes*. We defined four mutually exclusive groups based on the physician diagnoses, as documented in the Electronic Medical Records (EMR): ASD, ADHD only, other DDs only and neurotypical. Children in the ASD group included those ever diagnosed with autism ICD-9 code 299.00, 299.01; ICD-10 code 84.0), Asperger syndrome (ICD-9 code 299.80; ICD-10 code 84.5, 84.8), and/or pervasive developmental disorder not otherwise specified (ICD-9 code 299.90; ICD-10 code 84.9). When children had concurrent diagnoses, such as ASD and ADHD, or ASD and other DDs, they were still categorized as having ASD. Children in the ADHD only group included those with ADHD-related codes (ICD-9 codes 314.0-314.9 or ICD-10 codes F90.0-F90.9) but excluded those with ASD diagnosis. Children in the other DD only group included those who were diagnosed with mental, behavioral, and neurodevelopmental disorders (ICD-9 codes 290-319 or ICD-10 codes F01-F99) but excluded children with ASD and ADHD diagnosis. Neurotypical children were those that were never diagnosed with ASD, ADHD, and other DDs.

### Covariates

Based on the existing literature and our previous work [32, 35], we selected covariates for adjustment a priori. These included maternal pre-pregnancy BMI, diabetes status, maternal race/ethnicity, maternal age at delivery, parity (not including the index pregnancy), maternal education (high school or less vs. some college or more), smoking before or during pregnancy, child’s sex, preterm birth, and year of baby’s birth. Maternal pre-pregnancy weight and height were collected using a standardized questionnaire, which was used to calculate maternal BMI (BMI < 30, BMI ≥ 30). Maternal diabetes status was categorized into the following categories: 1) normal (i.e. without pregestational or gestational diabetes diagnosis); 2) gestational diabetes (ever diagnosed with diabetes mellitus complicating pregnancy); and 3) pregestational diabetes (ever diagnosed with diabetes). Neonates who were delivered ≥37 completed weeks of gestation were categorized as term and <37 weeks were considered preterm. Finally, in this minority cohort, self-reported maternal race/ethnicity was grouped into Black, White, Hispanic, and others (which included Asian, Pacific Islander, more than one race, and other).

### Statistical analysis

Maternal and child characteristics of the study children in the four outcome groups (ASD, ADHD only, other DD only and neurotypical) were compared using chi-square tests for categorical variables and analysis of variance test for continuous variables. Cord tryptophan metabolites were normalized using rank-based inverse normal transformation (similar to Z Scores) and were used for all subsequent analyses. The distribution of concentrations of cord tryptophan metabolites was compared across all the four groups. Pre-determined logistic regression models were applied to estimate the crude and adjusted associations between cord tryptophan metabolites and outcomes. After categorizing cord tryptophan metabolites into quartiles, adjusted logistic regression was used to examine the associations with the risk of ASD, ADHD, other DD with the neurotypical considered as the referent group. Our final model adjusted for maternal age at delivery, parity, maternal education, smoking, maternal race/ethnicity, maternal BMI, diabetes status, child sex, preterm birth and year of baby’s birth. We conducted stratified analyses using each stratum of covariates, including child’s sex, maternal race ethnicity and preterm birth to assess possible effect modification. We also conducted supplemental analysis to assess if any of the covariates (identified in this study or earlier studies [33, 36]) affected the cord tryptophan metabolites.

We performed the following sensitivity analyses: 1) using a stringent criterion of ASD diagnosis on two occasions, one of them being a visit to specialists such as developmental-behavioral pediatrician, pediatric neurologist, or child psychologist; 2) using a stringent criterion for neurotypical by further excluding children with Congenital Anomalies (740–759.9). All results are presented as odds ratio. Two-sided tests were used with a 0.05 significance level. Given the five metabolites that were considered in the analyses, we have also presented an adjusted P-value using Bonferroni correction (cutoff: 0.05/5 = 0.01). Data were analyzed using STATA version 13.0 (StataCorp, College Station, TX). Data described in the manuscript, code book, and analytic code is available upon request pending IRB review and approval.

## Results

Of 996 participants, the final sample included 326 neurotypical children, and 87 any ASD, 269 ADHD only and 314 other DD only group children (eFig. 1). The demographic and clinical characteristics of mothers and children are presented in Table 1 and have been documented in earlier studies in this cohort [35]. When compared to the mothers of neurotypical children, their counterparts in the ASD group were more likely to have had at least some college or more (49.43% vs 32.52%, *P* = 0.004), smoked 3 months prior or during pregnancy (20.69% vs 9.51%, *P* < 0.001) and had diabetes mellitus (18.39% vs 7.97%, *P* = 0.02). Children in the ASD group were more likely to be male (78.16% vs 38.34%, *P* < 0.001), born preterm (28.74% vs 8.28%, *P* < 0.001) and born during or after year 2007 (73.56% vs. 57.06%, *P* < 0.001). Similarly, mothers of children in the ADHD-only group, were more likely to have smoked 3 months prior or during pregnancy (22.30% vs 9.51%, *P* < 0.001), compared to the mothers of children in the neurotypical group. Children in the ADHD only group were more likely to be male (76.95% vs 38.34%, *P* < 0.001), born preterm (21.19% vs. 8.28%, *P* < 0.001) and born prior to year 2007 (43.87% vs. 57.06%, *P* = 0.001), compared to their neurotypical counterparts.

**Table 1:** Maternal and child characteristics according to child physician-diagnosed conditions.

Characteristics	Neurotypical (*n* = 326)	ASD (*n* = 87)	ADHD (*n* = 269)	Other DDs (*n* = 314)	*P* value
*Maternal age at birth (y)*					0.08
<20	33 (10.12)	0 (0.0)	26 (9.67)	21 (8.28)	
20-34	240 (73.62)	71 (81.61)	192 (71.38)	225 (71.66)	
≥35	53 (16.26)	16 (18.39)	51 (18.96)	63 (20.06)	
*Parity (%)*					0.81
0	135 (41.41)	40 (45.98)	111 (41.26)	126 (40.13)	
1 or more	191 (58.59)	47 (54.02)	158 (58.74)	188 (59.87)	
*Mother’s education (%)*					0.005
High school or less	220 (67.48)	44 (50.57)	192 (71.38)	207 (65.92)	
Some college or more	106 (32.52)	43 (49.43)	77 (28.62)	107 (34.08)	
*Maternal BMI (%)*					0.15
<30	267 (81.90)	65 (74.71)	201 (74.72)	241 (76.75)	
≥30	59 (18.10)	22 (25.29)	68 (25.28)	73 (23.25)	
*Diabetes (%)*					0.03
No	300 (92.02)	71 (81.61)	234 (86.99)	263 (83.76)	
Gestational diabetes mellitus	17 (5.21)	11 (12.64)	22 (8.18)	29 (9.24)	
Diabetes mellitus	9 (2.76)	5 (5.75)	13 (4.83)	22 (7.01)	
*Smoking during & 3 months prior to pregnancy (%)*					0.001
No	289 (88.65)	68 (78.16)	207 (76.95)	250 (79.62)	
Yes	31 (9.51)	18 (20.69)	60 (22.30)	62 (19.75)	
Missing	6 (1.84)	1 (1.15)	2 (0.74)	2 (0.64)	
Child					
*Sex (%)*					<0.001
Male	125 (38.34)	68 (78.16)	207 (76.95)	150 (47.77)	
Female	201 (61.66)	19 (21.84)	62 (23.05)	164 (52.23)	
*Race ethnicity (%)*					0.003
Black	222 (68.10)	43 (49.43)	170 (63.20)	198 (63.06)	
White	11 (3.37)	4 (4.60)	23 (8.55)	15 (4.78)	
Hispanic	64 (19.63)	29 (33.33)	59 (21.93)	84 (26.75)	
Other	29 (8.90)	11 (12.64)	17 (6.32)	17 (5.41)	
*Gestational age (%)*					<0.001
Term	299 (91.72)	62 (71.26)	212 (78.81)	246 (78.34)	
Preterm (<37 weeks)	27 (8.28)	25 (28.74)	57 (21.19)	68 (21.66)	
*Year of birth (%)*					<0.001
1999-2006	140 (42.94)	23 (26.44)	151 (56.13)	120 (38.22)	
2007-2013	186 (57.06)	64 (73.56)	118 (43.87)	194 (61.78)	
*Tryptophan pathway metabolites*					
Tryptophan, mean (SD)	−0.03 (1.02)	−0.12 (0.93)	0.16 (0.99)	−0.07 (0.99)	0.02
5-hydroxytryptophan, mean (SD)	−0.08 (0.92)	0.13 (0.8)	0.20 (0.99)	−0.07 (0.96)	<0.001
Serotonin, mean (SD)	0.02 (0.95)	0.16 (1.08)	0.04 (1.01)	−0.09 (1.01)	0.13
N-acetyltryptophan	−0.12 (1.02)	0.08 (1.02)	0.19 (1.04)	−0.06 (0.92)	0.001
5-methoxytryptophol, mean (SD)	0.11 (0.87)	−0.48 (1.09)	−0.14 (1.10)	0.15 (0.93)	<0.001

*ASD* autism spectrum disorder, *ADHD* attention-deficit/hyperactivity disorder, *DD* developmental disabilities.

A Bonferroni correction resulted in a significance level *P* < 0.01.

**Fig. 2:**
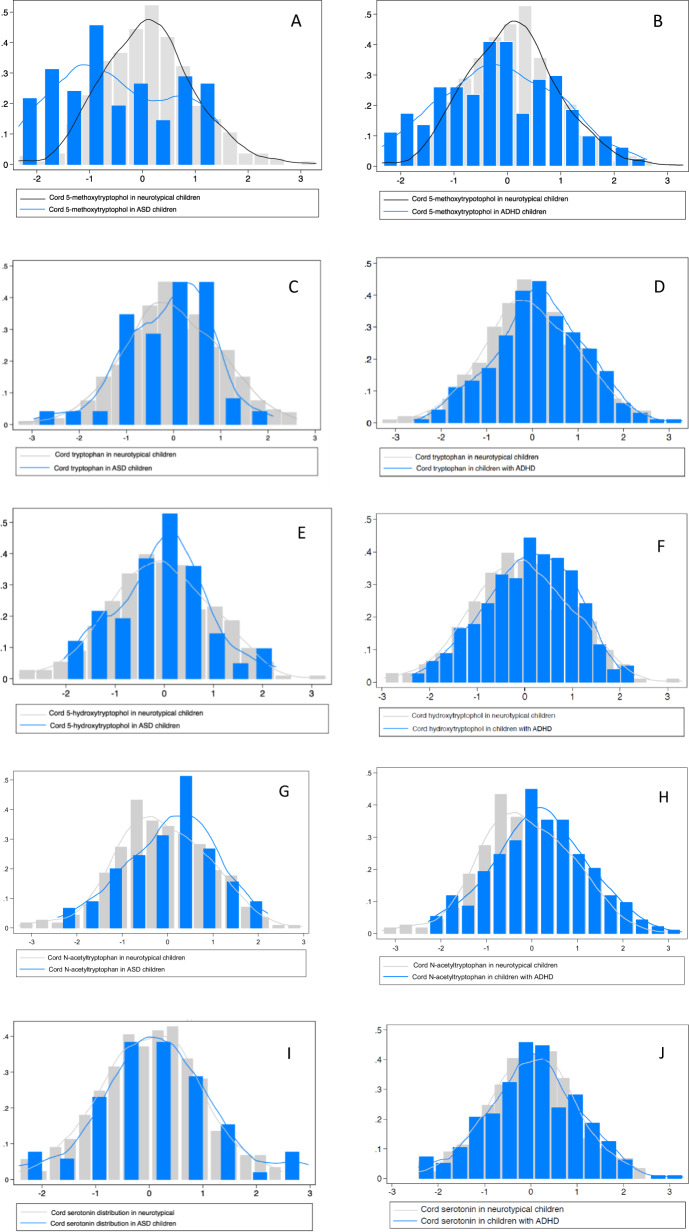
Distribution of cord tryptophan and its selected metabolites among those with ASD, ADHD, and other DD. **A** Cord 5-methoxytryptophol distribution in neurotypical and ASD children. **B** Cord 5-methoxytryptophol distribution in neurotypical and ADHD children. **C** Cord tryptophan distribution in neurotypical and ASD children. **D** Cord tryptophan distribution in neurotypical and ADHD children. **E** Cord 5-hydroxytryptophol distribution in neurotypical and ASD children. **F** Cord 5-hydroxytryptophol distribution in neurotypical and ADHD children. **G** Cord N-acetyltryptophan distribution in neurotypical and ASD children. **H** Cord N-acetyltryptophan distribution in neurotypical and ADHD children. **I** Cord serotonin distribution in neurotypical and ASD children. **J** Cord serotonin distribution in neurotypical and ADHD children. ASD autism spectrum disorder, ADHD attention-deficit/hyperactivity disorder.

eFigure 2 and eTable 1 present the correlation matrix of tryptophan metabolites (i.e. tryptophan, 5-HTP, N-acetyltryptophan, serotonin and 5-MTX). Cord 5-MTX was inversely correlated with cord tryptophan and N-acetlytryptophan in ASD and ADHD, and such correlation was not seen in neurotypical children. The distribution of tryptophan and its metabolites for children with ASD and ADHD are presented in Fig. 2. When compared to neurotypical children, the mean cord 5-MTX was significantly reduced in those who were later diagnosed with ASD (*P* < 0.001), evident by the 5-MTX distribution shifted slightly left (Fig. 2A). However, there was no difference in the distribution of other cord tryptophan metabolites in neurotypical children and those who were later diagnosed with ASD (*P* > 0.05) (Fig. 2C, E, G, I). The mean cord tryptophan metabolites in those subsequently diagnosed with ADHD was significantly different than neurotypical children for most of the metabolites except serotonin (Fig. 2, J). That is, mean tryptophan (*P* = 0.02), 5-HTP (*P* < 0.001) and N-acetyltryptophan (*P* < 0.001) were higher in those who were subsequently diagnosed with ADHD, as seen in the distribution of these metabolites shifted slightly right (Fig. 2D, F, H). On the other hand, mean 5-MTX was significantly lower among those subsequently diagnosed with ADHD (*P* = 0.02), with the distribution slightly shifted to the left (Fig. 2B).

### Tryptophan metabolites in cord blood and neurodevelopmental conditions: overall analyses

Table 2 presents the association between selected metabolites in the tryptophan pathway and subsequent risk of ASD, ADHD and other DD. Of these metabolites, higher cord 5-MTX levels were associated with a lower risk of ASD (aOR: 0.56, 95% CI: 0.41, 0.77), after adjusting for potential confounders. These findings were significant even after applying Bonferroni correction (*P* < 0.01). When stratified, those with cord 5-MTX above the cutoff (≥25th percentile), compared to those in the lowest quartile (<25th percentile), had a 73% reduction in the odds of ASD (aOR: 0.27, 95% CI: 0.14, 0.49). Further stratifying by quartiles showed consistent associations, with cord 5-MTX in higher quartiles having lower odds of subsequent ASD, when compared to the referent group (quartile 1). Cord tryptophan and other metabolites (5-HTP, N-acetyltryptophan, serotonin) were not associated with the subsequent risk of ASD.

**Table 2:** Association between cord 5-methoxytryptophol (5-MTX) and ASD (any), ADHD (without ASD), and other DD (without ASD).

			ASD (*n* = 87)			ADHD (*n* = 269)			Other DD (*n* = 314)		
	Total *n*	ND *n*	*N*	Crude OR (95% CI)	Adjusted^a^ OR (95% CI)	*n*	Crude OR (95% CI)	Adjusted^a^ OR (95% CI)	*n*	Crude OR (95% CI)	Adjusted^a^ OR (95% CI)
5 -MTX											
Continuous^b^	996	326	87	0.49 (0.37, 0.65)	0.56 (0.41, 0.77)	269	0.77 (0.65, 0.91)	0.79 (0.65, 0.96)	314	1.05 (0.88, 1.25)	1.09 (0.90, 1.31)
Quartiles											
Q1	249	60	44	Ref	Ref	89	Ref	Ref	56	Ref	Ref
Q2–Q4	747	266	43	0.22 (0.13, 0.37)	0.27 (0.14, 0.49)	180	0.46 (0.31, 0.67)	0.45 (0.29, 0.70)	258	1.04 (0.69, 1.55)	1.21 (0.78, 1.87)
Quartiles											
Q1	249	60	44	Ref	Ref	76	Ref	Ref	41	Ref	Ref
Q2	249	94	12	0.17 (0.09, 0.36)	0.19 (0.08, 0.44)	61	0.44 (0.28, 0.69)	0.42 (0.24, 0.72)	82	0.93 (0.58, 1.49)	1.03 (0.62, 1.70)
Q3	249	93	14	0.21 (0.10, 0.41)	0.29 (0.13, 0.66)	48	0.35 (0.22, 0.56)	0.31 (0.17, 0.54)	94	1.08 (0.68, 1.72)	1.40 (0.85, 2.32)
Q4	249	79	17	0.29 (0.15, 0.56)	0.34 (0.16, 0.75)	71	0.61 (0.38, 0.96)	0.66 (0.38, 1.13)	82	1.11 (0.69, 1.79)	1.24 (0.74, 2.06)
Tryptophan											
Continuous^b^	996	326	87	0.92 (0.73, 1.16)	0.90 (0.67, 1.22)	269	1.21 (1.03, 1.42)	1.25 (1.03, 1.51)	314	0.96 (0.83, 1.12)	0.93 (0.79, 1.10)
Quartiles											
Q1	249	83	26	Ref	Ref	53	Ref	Ref	87	Ref	Ref
Q2–Q4	747	243	61	0.80 (0.48, 1.35)	0.77 (0.40, 1.46)	216	1.39 (0.94, 2.06)	1.59 (1.01, 2.50)	227	0.89 (0.63, 1.27)	0.81 (0.56, 1.19)
Quartiles											
Q1	249	83	26	Ref	Ref	53	Ref	Ref	87	Ref	Ref
Q2	249	91	14	0.49 (0.24, 1.00)	0.49 (0.21, 1.14)	65	1.12 (0.70, 1.79)	1.35 (0.78, 2.32)	79	0.83 (0.54, 1.27)	0.79 (0.50, 1.24)
Q3	249	72	34	1.51 (0.83, 2.75)	1.33 (0.63, 2.81)	69	1.50 (0.93, 2.42)	1.72 (0.98, 3.01)	74	0.98 (0.63, 1.53)	0.83 (0.51, 1.34)
Q4	249	80	13	0.52 (0.25, 1.08)	0.56 (0.24, 1.33)	82	1.61 (1.01, 2.55)	1.74 (1.02, 2.98)	74	0.88 (0.57, 1.37)	0.83 (0.52, 1.33)
5-hydroxytryptophan											
Continuous^b^	996	326	87	1.27 (0.98, 1.64)	1.28 (0.96, 1.73)	269	1.36 (1.14, 1.62)	1.32 (1.08. 1.61)	314	1.01 (0.86, 1.20)	0.94 (0.79, 1.12)
Quartiles											
Q1	250	92	18	Ref	Ref	49	Ref	Ref	91	Ref	Ref
Q2-Q4	746	234	69	1.51 (0.85, 2.67)	1.86 (0.94, 3.69)	220	1.77 (1.19, 2.61)	1.94 (1.24, 3.05)	223	0.96 (0.68, 1.36)	0.89 (0.62, 1.28)
Quartiles											
Q1	250	92	18	Ref	Ref	49	Ref	Ref	73	Ref	Ref
Q2	248	79	24	1.55 (0.79, 3.07)	2.15 (0.94, 4.87)	68	1.62 (1.01, 2.60)	2.04 (1.18, 3.53)	66	0.99 (0.64, 1.51)	0.96 (0.61, 1.52)
Q3	249	85	25	1.50 (0.77, 2.95)	1.81 (0.80, 4.08)	65	1.44 (0.89, 2.31)	1.59 (0.92, 2.73)	59	0.88 (0.58, 1.35)	0.85 (0.54, 1.34)
Q4	249	70	20	1.46 (0.72, 2.97)	1.65 (0.71, 3.84)	87	2.33 (1.46, 3.73)	2.29 (1.33, 3.95)	63	1.04 (0.67, 1.61)	0.85 (0.53, 1.36)
Serotonin											
Continuous^b^	996	326	87	1.16 (0.91, 1.47)	1.18 (0.89, 1.58)	269	1.03 (0.87, 1.21)	1.10 (0.91, 1.34)	314	0.89 (0.76, 1.04)	0.87 (0.73, 1.04)
Quartiles											
Q1	249	75	17	Ref	Ref	60	Ref	Ref	97	Ref	Ref
Q2–Q4	747	251	70	1.23 (0.68, 2.22)	1.36 (0.65, 2.86)	209	1.04 (0.71, 1.53)	1.12 (0.72, 1.76)	217	0.67 (0.47, 0.95)	0.65 (0.45, 0.96)
Quartiles											
Q1	249	75	17	Ref	Ref	60	Ref	Ref	97	Ref	Ref
Q2	249	81	23	1.25 (0.62, 2.53)	1.40 (0.59, 3.31)	64	0.99 (0.62, 1.58)	0.97 (0.56, 1.68)	81	0.77 (0.50, 1.19)	0.76 (0.48, 1.20)
Q3	249	95	22	1.02 (0.51, 2.06)	1.22 (0.51, 2.92)	74	0.97 (0.62, 1.54)	1.04 (0.61, 1.79)	58	0.57 (0.30, 0.74)	0.48 (0.30, 0.78)
Q4	249	75	25	1.47 (0.73, 2.94)	1.46 (0.62, 3.44)	71	1.18 (0.74, 1.89)	1.41 (0.82, 2.43)	78	0.80 (0.52, 1.25)	0.76 (0.47, 1.22)
N-acetyltryptophan											
Continuous^b^	996	326	87	1.21 (0.96, 1.53)	1.06 (0.81, 1.39)	269	1.34 (1.14, 1.57)	1.27 (1.05, 1.53)	314	1.06 (0.91, 1.25)	0.97 (0.81, 1.15)
Quartiles											
Q1	249	99	17	Ref	Ref	56	Ref	Ref	77	Ref	Ref
Q2–Q4	747	227	70	1.80 (1.01, 3.21)	1.51 (0.77, 2.96)	213	1.66 (1.14, 2.42)	1.78 (1.14, 2.77)	237	1.34 (0.95, 1.90)	1.14 (0.79, 1.66)
Quartiles											
Q1	249	99	17	Ref	Ref	56	Ref	Ref	77	Ref	Ref
Q2	249	81	20	1.44 (0.71, 2.93)	1.20 (0.52, 2.80)	59	1.29 (0.81, 2.06)	1.60 (0.93, 2.76)	89	1.41 (0.93, 2.16)	1.21 (0.77, 1.90)
Q3	249	68	27	2.31 (1.17, 4.57)	2.56 (1.13, 5.81)	74	1.92 (1.21, 3.06)	2.09 (1.21, 3.61)	80	1.51 (0.97, 2.35)	1.36 (0.85, 2.17)
Q4	249	78	23	1.72 (0.86, 3.44)	1.15 (0.51, 2.59)	80	1.81 (1.15, 2.85)	1.69 (0.99, 2.88)	68	1.12 (0.72, 1.74)	0.87 (0.54, 1.41)

*ASD* autism spectrum disorder, *ADHD* attention-deficit/hyperactivity disorder, *DD* developmental disabilities, *5-MTX* 5-methoxytryptophol.

^a^Adjusted for maternal age, maternal education, parity, smoking status, diabetes, BMI, race/ethnicity (black, white, Hispanic, others), preterm status and year of birth.

^b^Cord 5-MTX was normalized using the rank-based inverse normal transformation, which is similar to *Z* Scores.

A Bonferroni correction resulted in a significance level of *P* < 0.01.

Next, we assessed the association between cord tryptophan and its metabolites with the subsequent risk of ADHD. When considered as a continuous variable, one unit increase in tryptophan (aOR: 1.25, 95% CI: 1.03, 1.51), 5-HTP (aOR: 1.32, 95% CI: 1.08, 1.61) and N-acetyltryptophan (aOR: 1.27, 95% CI: 1.05, 1.53) were associated with an increased risk of subsequent ADHD, after accounting for potential confounders. These findings were mostly consistent when the metabolites were further stratified by quartiles, as well as when the metabolites in the lowest quartile (<25th percentile) were compared against those above this cutoff (≥25th percentile). Cord blood serotonin, however, was not associated with the risk of ADHD. In contrast to the other tryptophan metabolites, 5-MTX was inversely associated with ADHD risk. That is, when 5-MTX was considered as a continuous variable, higher cord 5-MTX levels were associated with a lower odds of ADHD (aOR: 0.79, 95% CI: 0.65, 0.91), after adjusting for potential confounders. Further stratifying cord 5-MTX ( < 25th percentile vs. ≥25th percentile) showed a consistent inverse association with ADHD (aOR: 0.45, 95% CI: 0.29, 0.70). The only exception was that the children that had highest 5-MTX levels (quartile 4), whose odds of ADHD was not significantly different compared to the referent group (quartile 1). There was no association between 5-MTX and other DDs, when 5-MTX was considered as a continuous or categorical variable.

### Tryptophan metabolites and neurodevelopmental conditions: subgroup analyses

**Fig. 3:**
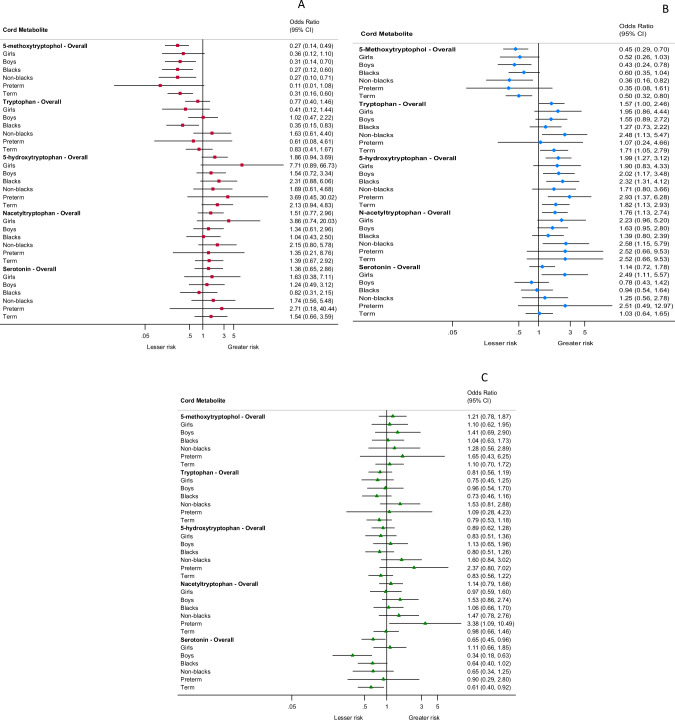
Forest plots summarizing the subgroup analysis of association between select tryptophan metabolites with risk of ASD, ADHD and other DD. **A** Association between cord tryptophan metabolites and risk of ASD, overall and across sub-groups. **B** Association between cord tryptophan metabolites and risk of ADHD, overall and across sub-groups. **C** Association between cord tryptophan metabolites and risk of other DD, overall and across sub-groups. ASD autism spectrum disorder, ADHD attention-deficit/hyperactivity disorder, DD developmental disabilities.

We repeated the main analysis by restricting the sample to high-risk sub-groups for each of the metabolites and risk of ASD (Fig. 3A), ADHD (Fig. 3B) and other DD (Fig. 3C) (eTables 2–6). An inverse association between cord 5-MTX and risk of ASD was observed in all sub-groups, except boys (aOR: 0.36, 95% CI: 0.12, 1.10) and preterm babies (aOR: 0.11, 95% CI: 0.01, 1.08). For other metabolites (i.e. tryptophan, 5-HTP, N-acetyltrypophan and serotonin), there was no association between their levels and subsequent risk of ASD in any of these high-risk sub-groups. Similarly, higher cord 5-MTX was associated with a lower risk of ADHD for most of the sub-groups except boys (aOR: 0.52, 95% CI: 0.26, 1.03), preterms (aOR: 0.35, 95% CI: 0.08, 1.61) and blacks (aOR: 0.60, 95% CI: 0.35, 1.04). The risk of ADHD was elevated among certain sub-groups for cord tryptophan, 5-HTP, N-acetyltryptophan and serotonin, but not in all sub-groups. There were no significant associations between these sub-groups and other DD, for any of the metabolites examined in this study.

We further investigated whether certain covariates influenced 5-MTX concentrations. As reported in eTable 7, the covariates, including Tylenol score [33] and branched chain amino acids [36] (shown to be associated with ASD in earlier studies) did not influence cord 5-MTX levels; although, preterm birth (*P* = 0.04) and maternal diabetes or obesity (*P* = 0.04) was significantly associated with lower cord 5-MTX levels. Next, we sequentially added covariates to the model, but none of these affected the association between 5-MTX and ASD (eTable 8). The results of the sensitivity analyses demonstrated consistent associations in directionality when a stringent ASD criterion and comparator were applied (eTables 9 and 10).

## Discussion

This prospective cohort study revealed that cord plasma 5-MTX, a pineal gland metabolite in the tryptophan pathway, is inversely associated with the risk of ASD and ADHD. Further, cord plasma tryptophan and other metabolites examined in this study were associated with the subsequent risk of ADHD, but not ASD. However, cord serotonin was not associated with ASD or ADHD. Most of our findings remained statistically significant after additional sensitivity analyses, including sequential adjustment of potential confounders, and subgroup analyses.

### 5-MTX and risk of ASD and ADHD

Tryptophan, an essential amino acid, is metabolized along two main pathways: hydroxyindole and kynurenine. Although only a small fraction of tryptophan enters the former pathway, it is still important for the generation of several metabolites considered in this report. 5-MTX is part of this complex network of hydroxyindole metabolism in the central nervous system and is synthesized from its precursors serotonin and melatonin (Fig. 1) [37]. Until now, research on this pineal gland metabolite, although minimal, has primarily focused on diurnal rhythm. Our study supports the hypothesis that 5-MTX may possess neuroprotective abilities with higher levels in cord blood associated with a lower risk of both ASD and ADHD. This further suggests a shared etiology between ASD and ADHD [29]. Our findings are in line with a recent clinical trial, which showed that 5-MTX is significantly increased among patients with MDD that responded favorably to selective serotonin reuptake inhibitors [20, 21]. Our study did not find an association between other tryptophan metabolites in cord blood and risk of ASD. Some of our findings are similar to the above-mentioned trial that also assessed tryptophan, 5-HTP, but did not find an effect on MDD based on selective serotonin reuptake inhibitor treatment [20, 21]. Other than these, to our knowledge, very few studies have assessed this prospective relationship between cord plasma 5-MTX and the risk of ASD and ADHD and hence, it is difficult to compare our findings with others. Nevertheless, additional studies with larger sample sizes are needed to explore the associations between tryptophan metabolites and ASD.

Sleep disorder is one of the most common co-morbidities in children with ASD and ADHD [1, 38–42]. A recent study showed that sleep problems were noted as early as 6-12 months in children, much earlier than the child’s ASD diagnosis [43], suggesting that sleep disorders and possibly underlying pathological mechanisms are present at birth and/or during the early postnatal period. Studies that have assessed melatonin (a precursor of 5-MTX and a metabolite that possesses similar properties [15]) is shown to be altered in ADHD [44–46] and ASD [39, 47–55] and is associated with autistic social communication impairments [38, 39, 55, 56]. Further, ASD is now considered a condition with impaired synchrony of motor, emotional and relational rhythms and associated imbalance in chronobiotics like melatonin [57]. Taken together the available literature and our findings, 5-MTX level and related metabolic pathway may be altered in children with ASD, which may be seen at birth as measured in cord plasma.

From a developmental perspective, it is known that precursors of 5-MTX, serotonin and melatonin can cross the placental barrier [58–62] and can also be produced endogenously in the placenta [63–66]. Both serotonin and melatonin have important roles in prenatal brain development [63, 64, 67–69]. There is emerging evidence that melatonin is involved during early development through its influence on placenta, developing neurons and glia, and its role in ontogenic establishment of diurnal rhythms [48, 55]. While the source of cord 5-MTX is unclear, it could be speculated that possible malfunctioning of the pathway in mothers could have resulted in altered exposure to the fetus in utero [48, 54], but more research is needed in humans to understand the underlying mechanism An alternative hypothesis is that lower levels of 5-MTX, a potent antioxidant that possesses immunomodulatory properties on cytokine secretion, may have prompted abnormal immune response and possibly altered the brain development [70–72]. Several genes in the tryptophan pathway seem to be implicated in ASD, but additional studies are needed to understand the role of genes involved in the synthesis, transport, and degradation of 5-MTX [73].

### Tryptophan metabolites and risk of ADHD

Our findings are consistent with earlier studies that showed elevated serum tryptophan levels in children with ADHD [13, 74]. Urinary tryptophan may also be altered with higher levels of tryptophan reported in children with ADHD [75]. Altered tryptophan levels have shown to be associated with ADHD symptoms such as inattention, response time and its variability as well as increases in errors of commission [76]. The underlying mechanism for the relationship between tryptophan and ADHD is not well-understood; but, one study suggested diminished tryptophan transport across fibroblast cell membranes and possibly across the blood–brain barrier may be observed in ADHD [77]. Another study hypothesized that tryptophan metabolites are altered resulting in anomalous glial function, which is often noted in ADHD [78]. Our findings, in the context of the existing evidence, suggest that the altered availability of tryptophan and its metabolites may not be an isolated occurrence. Rather, it provides evidence to support the hypothesis that there may be disruption in the tryptophan pathway in ADHD, as evidenced by alterations in most of the metabolites considered in this study. If our findings are consistent with future studies, cord tryptophan and its metabolites can be considered as a biomarker of ADHD, as anomalies in these metabolites may be observed much before the manifestation of behavioral symptoms.

Of the metabolites considered in this study, cord serotonin was not associated with the risk of ASD and ADHD. While the reason for this finding is unclear, it is possible that a few factors might explain the differences. First, most of the evidence on hyperserotonemia comes from studies conducted on children and adults diagnosed with ASD and/or ADHD [11, 79, 80]. To the best of our knowledge, very few studies have measured serotonin at birth and preliminary evidence suggests that the levels of these neurotransmitters are lower at birth and increase rapidly during infancy [81]. While our findings suggest that serotonin levels did not vary at birth, it is unclear if the rapid increase during infancy is differential by subsequent diagnosis. Second, the race/ethnicity of the participants is known to influence serotonin levels, with Blacks and Latinos having higher platelet serotonin [79]. Third, it is possible that other aspects of serotonin metabolism, including serotonin transport, could shed additional light on ASD and ADHD etiology; however, this study was not designed to assess that association. Nevertheless, additional longitudinal studies are needed to further understand the association between cord blood serotonin in the context of subsequent risk of ASD and ADHD.

### Limitations

Our findings should be considered in the context of the following limitations. First, this study included one-time measurement of cord tryptophan and its metabolites at birth and did not measure it subsequently to understand how they varied with age. The fact that some of the metabolites showed an association with incident ASD and ADHD in childhood is an important finding and worth additional investigation. Our study only examined certain metabolites in the pathway; future studies should consider melatonin, another important chronobiotic in the pathway, to simultaneously assess their inter-relationships and joint association with ASD and ADHD. Further, current metabolomics platform generates relative intensity rather than actual concentration of the metabolites. Second, the study children were characterized as neurotypical vs. having neurodevelopmental disability (i.e., ASD or ADHD or other DD) based on physician diagnosis, as documented in the EMR and this could have led to some potential misclassification. However, this misclassification may not be differential given the prospective study design and objective lab measurements of cord tryptophan and its metabolites, which were conducted by laboratory personnel who were unaware of the case status. As such, any misclassification would have biased the results towards the null. Third, other DD group in our analysis included a number of conditions due to disabilities in physical, learning, language, or behavioral areas. While it would have been insightful to assess the association between cord 5-MTX and each of these specific conditions, summarized under the broad other DD category, our study was not powered to do so. Fourth, this study was limited to those who continued to seek pediatric care at the BMC. While selection bias is a concern, this is assuaged by that the baseline characteristics of the participants initially enrolled in the study and remaining BBC sample is comparable. Fifth, sunlight is known to strongly influence circadian rhythm. As shown in supplemental eFig. 3, we found that the distribution of 5-MTX is similar between the births happening during daytime or nighttime, consistent with less established circadian rhythm in the fetal brain. Besides, there was no difference in the percentage of delivery between happening at daytime vs nighttime for any of the outcomes (eTable 11, *P* = 0.68). Sixth, because of our observational study design, we cannot exclude the possibility of residual confounding. Finally, considering that our study was conducted in a high-risk population, a predominantly urban, low-income, racially diverse, with a high proportion of preterm births, caution should be exercised when extrapolating our findings to other populations with different characteristics.

## Conclusions

In summary, this is the first prospective birth cohort study that linked multiple metabolites of tryptophan as measured at birth with the risk of ASD, ADHD, and other DD. We found that 5-MTX in cord blood at birth was associated with subsequent ASD and ADHD diagnosis. Multiple tryptophan metabolites considered in this study including tryptophan, 5-hydroxytryptophan, and N-acetyltryptophan were associated with the risk of ADHD, possibly suggesting a global disruption in the tryptophan pathway. This study should be regarded as hypothesis-generating, since very little is known about the role of 5-MTX in humans. Additional research is needed to study the association of 5-MTX at different time points with the evolution of clinical features of ASD and ADHD to better understand the temporal relation and pathophysiological significance of 5-MTX. Future studies can also explore the combination of metabolites on ASD and ADHD, since some research suggest this may lead to good discrimination between individuals with vs. without ADHD [10, 54].

## Supplementary information

Supplemental Tables and Figures
