# Supplementary material for: Association between cord blood metabolites in tryptophan pathway and childhood risk of autism spectrum disorder and attention-deficit hyperactivity disorder
Source: Transl Psychiatry. 2022 Jul 9;12:270. doi: 10.1038/s41398-022-01992-0 (PMC9271093; doi:10.1038/s41398-022-01992-0)
Supplement: Supplementary file 1 — Supplemental Tables and Figures [file 41398_2022_1992_MOESM1_ESM.docx]

eFigure 1: Flowchart of sample inclusion and exclusion

eFigure 2: Correlation matrices between cord tryptophan and its metabolites in neurotypical children and those with ASD, ADHD and other DD

eFigure 3: Distribution of 5-MTX by time of birth

eTable 1: Association between cord 5-methoxytryptophol (5-MTX) and ASD, ADHD, and other DD

eTable 2: Association between cord 5-methoxytryptophol (5-MTX) and ASD, ADHD, and other DD – Only in Boys

eTable 3: Association between cord 5-methoxytryptophol (5-MTX) and ASD, ADHD, and other DD – Only in girls

eTable 4: Association between cord 5-methoxytryptophol (5-MTX) and ASD, ADHD, and other DD – Only in Blacks

eTable 5: Association between cord 5-methoxytryptophol (5-MTX) and ASD, ADHD, and other DD – Only in non-blacks

eTable 6: Association between cord 5-methoxytryptophol (5-MTX) and ASD, ADHD, and other DD – Only in Preterm

eTable 7: Factors associated with cord 5-methoxytryptophol

eTable 8: Sequential modeling to identify influential covariates on the association between 5-MTX and ASD in total sample

eTable 9: Sensitivity Analysis: Association between 5-methoxytryptophol and ASD among those that were diagnosed at least twice, including a visit to a specialist

eTable 10: Sensitivity Analysis: Association between 5-methoxytryptophol and ASD, ADHD and other DD using a stringent comparator group

eTable 11: Neurodevelopmental conditions by time of birth

## eFigure 1: Flowchart of sample inclusion and exclusion

**n=996** children with cord plasma tryptophan and its metabolites measurements

- 87 children with any ASD diagnosis
- 269 children with any ADHD diagnosis excluding ASD
- 314 children with any other developmental disabilities diagnosis excluding ASD and ADHD
- 326 children without any developmental disabilities diagnosis

**n=3,165** mother-infant dyads with EMR records of well-child and specialty visits

- 124 children with any ASD diagnosis
- 448 children with any ADHD diagnosis excluding ASD
- 1153 children with any other developmental disabilities diagnosis excluding ASD and ADHD
- 1440 children without any developmental disabilities diagnosis

**n=2,169**

Excluded because of missing cord tryptophan and its metabolites data

## Figure 2: Correlation matrices between cord tryptophan and its metabolites in neurotypical children (A) and those with ASD (B), ADHD (C) and other DD (D)

BA

D

C

A

## eFigure 3: Distribution of 5-Methoxytryptophol by time of birth

## eTable 1: Correlation of cord tryptophan and its metabolites

|  | Tryptophan | N-acetyltryptophan | 5-hydroxytryptophan | Serotonin | 5-methoxytryptophol |
| --- | --- | --- | --- | --- | --- |
| **Overall** |  |  |  |  |  |
| Tryptophan | 1.0 | 0.6361 | 0.5171 | 0.4620 | -0.0111 |
| N-acetyltryptophan | 0.6361 | 1.0 | 0.4123 | 0.3344 | -0.0330 |
| 5-hydroxytryptophan | 0.5171 | 0.4123 | 1.0 | 0.3095 | -0.0166 |
| Serotonin | 0.4620 | 0.3344 | 0.3095 | 1.0 | 0.0588 |
| 5-methoxytryptophol | -0.0111 | -0.0330 | -0.0166 | 0.0588 | 1.0 |
| **Neurotypical** |  |  |  |  |  |
| Tryptophan | 1.0 | 0.6316 | 0.4896 | 0.4441 | 0.3745 |
| N-acetyltryptophan | 0.6316 | 1.0 | 0.3821 | 0.2964 | 0.0395 |
| 5-hydroxytryptophan | 0.4896 | 0.3821 | 1.0 | 0.2282 | -0.0527 |
| Serotonin | 0.4441 | 0.2964 | 0.2282 | 1.0 | 0.0249 |
| 5-methoxytryptophol | 0.0591 | 0.0395 | -0.0527 | 0.0249 | 1.0 |
| **ASD** |  |  |  |  |  |
| Tryptophan | 1.0 | 0.6525 | 0.5891 | 0.5199 | -0.3959 |
| N-acetyltryptophan | 0.6525 | 1.0 | 0.4140 | 0.3488 | -0.2140 |
| 5-hydroxytryptophan | 0.5891 | 0.4140 | 1.0 | 0.4196 | -0.1874 |
| Serotonin | 0.5199 | 0.3488 | 0.4196 | 1.0 | -0.0852 |
| 5-methoxytryptophol | -0.3959 | -0.2140 | -0.1872 | -0.0852 | 1.0 |
| **ADHD** |  |  |  |  |  |
| Tryptophan | 1.0 | 0.6336 | 0.5332 | 0.4617 | -0.0427 |
| N-acetyltryptophan | 0.6336 | 1.0 | 0.4274 | 0.3415 | -0.0623 |
| 5-hydroxytryptophan | 0.5332 | 0.4274 | 1.0 | 0.3161 | 0.0356 |
| Serotonin | 0.4617 | 0.3415 | 0.3161 | 1.0 | 0.0720 |
| 5-methoxytryptophol | -0.0427 | -0.0623 | 0.0356 | 0.0720 | 1.0 |
| **Other DD** |  |  |  |  |  |
| Tryptophan | 1.0 | 0.6366 | 0.5041 | 0.4719 | 0.0849 |
| N-acetyltryptophan | 0.6366 | 1.0 | 0.3998 | 0.3645 | 0.0495 |
| 5-hydroxytryptophan | 0.5041 | 0.3398 | 1.0 | 0.3393 | 0.0849 |
| Serotonin | 0.4719 | 0.3645 | 0.3393 | 1.0 | 0.1721 |
| 5-methoxytryptophol | 0.0849 | 0.0495 | 0.0849 | 0.1721 | 1.0 |

## eTable 2: Association between cord 5-methoxytryptophol (5-MTX) and ASD, ADHD, and other DD – Only in Boys

|  |  |  | ASD | | | ADHD | | | Other DD | | |
| --- | --- | --- | --- | --- | --- | --- | --- | --- | --- | --- | --- |
|  | Total n | ND n | n | Crude | Adjusted^1^ | n | Crude | Adjusted^1^ | n | Crude | Adjusted^1^ |
| **5-MTX** |  |  |  |  |  |  |  |  |  |  |  |
| Continuous | 550 | 125 | 68 | 0.62 (0.45, 0.85) | 0.64 (0.45, 0.92) | 207 | 0.89 (0.72, 1.10) | 0.91 (0.72, 1.16) | 150 | 1.19 (0.91, 1.55) | 1.20 (0.90, 1.59) |
| Quartiles |  |  |  |  |  |  |  |  |  |  |  |
| Q1 | 138 | 22 | 29 | Ref | Ref | 66 | Ref | Ref | 21 | Ref | Ref |
| Q2 | 137 | 38 | 12 | 0.24 (0.10, 0.56) | 0.24 (0.09, 0.67) | 47 | 0.41 (0.22, 0.79) | 0.38 (0.19, 0.78) | 40 | 1.10 (0.52, 2.32) | 1.15 (0.51, 2.60) |
| Q3 | 138 | 40 | 13 | 0.25 (0.11, 0.57) | 0.33 (0.12, 0.88) | 36 | 0.30 (0.16, 0.58) | 0.24 (0.11, 0.50) | 49 | 1.28 (0.62, 2.66) | 1.47 (0.66, 3.28) |
| Q4 | 137 | 25 | 14 | 0.42 (0.18, 1.00) | 0.38 (0.14, 1.04) | 58 | 0.77 (0.39, 1.33) | 0.81 (0.39, 1.68) | 40 | 1.68 (0.77, 3.65) | 1.72 (0.74, 4.02) |
| Quartiles |  |  |  |  |  |  |  |  |  |  |  |
| Q1 | 138 | 22 | 29 | Ref | Ref | 66 | Ref | Ref | 21 | Ref | Ref |
| Q2-Q4 | 412 | 103 | 39 | 0.29 (0.15, 0.56) | 0.31 (0.14, 0.70) | 141 | 0.46 (0.26, 0.79) | 0.43 (0.24, 0.78) | 129 | 1.31 (0.68, 2.52) | 1.41 (0.69, 2.90) |
| **Tryptophan** |  |  |  |  |  |  |  |  |  |  |  |
| Continuous | 550 | 125 | 68 | 0.98 (0.72, 1.35) | 1.01 (0.70, 1.46) | 207 | 1.26 (1.00, 1.58) | 1.31 (1.02, 1.68) | 150 | 0.92 (0.71, 1.20) | 0.91 (0.68, 1.21) |
| Quartiles |  |  |  |  |  |  |  |  |  |  |  |
| Q1 | 138 | 33 | 20 | Ref | Ref | 42 | Ref | Ref | 43 | Ref | Ref |
| Q2 | 137 | 37 | 12 | 0.54 (0.23, 1.26) | 0.70 (0.26, 1.87) | 47 | 1.00 (0.53, 1.89) | 1.16 (0.60, 2.26) | 41 | 0.85 (0.45, 1.60) | 0.99 (0.51, 1.94) |
| Q3 | 138 | 26 | 23 | 1.46 (0.66, 3.21) | 1.70 (0.66, 4.39) | 55 | 1.66 (0.87, 3.19) | 1.85 (0.91, 3.76) | 34 | 1.00 (0.51, 1.99) | 1.09 (0.52, 2.30) |
| Q4 | 137 | 29 | 13 | 0.74 (0.31, 1.74) | 0.87 (0.32, 2.34) | 63 | 1.71 (0.91, 3.22) | 1.83 (0.93, 3.60) | 32 | 0.85 (0.43, 1.67) | 0.82 (0.39, 1.69) |
| Quartiles |  |  |  |  |  |  |  |  |  |  |  |
| Q1 | 138 | 33 | 20 | Ref | Ref | 42 | Ref | Ref | 43 | Ref | Ref |
| Q2-Q4 | 412 | 92 | 48 | 0.86 (0.45, 1.66) | 1.02 (0.47, 2.22) | 165 | 1.41 (0.84, 2.38) | 1.55 (0.89, 2.72) | 107 | 0.89 (0.52, 1.52) | 0.96 (0.54, 1.70) |
| **5-** **hydroxytryptophan** |  |  |  |  |  |  |  |  |  |  |  |
| Continuous | 550 | 125 | 68 | 1.33 (0.98, 1.82) | 1.40 (0.98, 2.01) | 207 | 1.44 (1.14, 1.82) | 1.38 (1.07, 1.79) | 150 | 1.02 (0.79, 1.32) | 0.93 (0.70, 1.22) |
| Quartiles |  |  |  |  |  |  |  |  |  |  |  |
| Q1 | 138 | 40 | 17 | Ref | Ref | 39 | Ref | Ref | 42 | Ref | Ref |
| Q2 | 138 | 28 | 17 | 1.43 (0.62, 3.27) | 1.58 (0.61, 4.11) | 49 | 1.79 (0.95, 3.41) | 1.95 (0.99, 3.85) | 44 | 1.50 (0.79, 2.84) | 1.46 (0.73, 2.91) |
| Q3 | 137 | 35 | 17 | 1.14 (0.51, 2.57) | 1.15 (0.45, 2.95) | 53 | 1.55 (0.84, 2.87) | 1.67 (0.87, 3.20) | 32 | 0.87 (0.46, 1.66) | 0.90 (0.46, 1.79) |
| Q4 | 137 | 22 | 17 | 1.82 (0.78, 4.25) | 2.19 (0.81, 5.93) | 66 | 3.08 (1.60, 5.92) | 2.65 (1.31, 5.34) | 32 | 1.39 (0.69, 2.77) | 1.07 (0.50, 2.30) |
| Quartiles |  |  |  |  |  |  |  |  |  |  |  |
| Q1 | 138 | 40 | 17 | Ref | Ref | 39 | Ref | Ref | 42 | Ref | Ref |
| Q2-Q4 | 412 | 85 | 51 | 1.41 (0.73, 2.75) | 1.54 (0.72, 3.34) | 168 | 2.03 (1.21, 3.38) | 2.02 (1.17, 3.48) | 108 | 1.21 (0.72, 2.03) | 1.13 (0.65, 1.96) |
| **Serotonin** |  |  |  |  |  |  |  |  |  |  |  |
| Continuous | 550 | 125 | 68 | 1.15 (0.86, 1.54) | 1.35 (0.95, 1.93) | 207 | 1.03 (0.82, 1.29) | 1.12 (0.89, 1.43) | 150 | 0.71 (0.55, 0.91) | 0.68 (0.51, 0.90) |
| Quartiles |  |  |  |  |  |  |  |  |  |  |  |
| Q1 | 138 | 23 | 14 | Ref | Ref | 47 | Ref | Ref | 54 | Ref | Ref |
| Q2 | 137 | 36 | 14 | 0.64 (0.26, 1.58) | 0.79 (0.27, 2.34) | 47 | 0.64 (0.33, 1.24) | 0.59 (0.29, 1.21) | 40 | 0.47 (0.24, 0.92) | 0.39 (0.19, 0.80) |
| Q3 | 138 | 37 | 19 | 0.84 (0.36, 2.00) | 1.51 (0.50, 4.56) | 52 | 0.69 (0.36, 1.32) | 0.64 (0.31, 1.30) | 30 | 0.35 (0.17, 0.69) | 0.30 (0.14, 0.64) |
| Q4 | 137 | 29 | 21 | 1.19 (0.50, 2.84) | 1.74 (0.60, 5.06) | 61 | 1.03 (0.53, 2.00) | 1.22 (0.59, 2.50) | 26 | 0.38 (0.19, 0.78) | 0.32 (0.15, 0.70) |
| Quartiles |  |  |  |  |  |  |  |  |  |  |  |
| Q1 | 146 | 23 | 14 | Ref | Ref | 47 | Ref | Ref | 54 | Ref | Ref |
| Q2-Q4 | 404 | 102 | 54 | 0.87 (0.41, 1.83) | 1.24 (0.49, 3.12) | 160 | 0.77 (0.44, 1.34) | 0.78 (0.43, 1.42) | 96 | 0.40 (0.23, 0.70) | 0.34 (0.18, 0.63) |
| **N-acetyltryptophan** |  |  |  |  |  |  |  |  |  |  |  |
| Continuous | 550 | 125 | 68 | 1.12 (0.84, 1.48) | 1.00 (0.72, 1.38) | 207 | 1.29 (1.04, 1.59) | 1.29 (1.02, 1.62) | 150 | 0.99 (0.76, 1.29) | 0.95 (0.71, 1.27) |
| Quartiles |  |  |  |  |  |  |  |  |  |  |  |
| Q1 | 138 | 40 | 15 | Ref | Ref | 48 | Ref | Ref | 35 | Ref | Ref |
| Q2 | 137 | 28 | 17 | 1.62 (0.69, 3.77) | 1.25 (0.47, 3.36) | 42 | 1.25 (0.66, 2.36) | 1.35 (0.68, 2.69) | 50 | 2.04 (1.07, 3.90) | 2.10 (1.05, 4.21) |
| Q3 | 138 | 27 | 17 | 1.68 (0.72, 3.92) | 1.58 (0.58, 4.30) | 53 | 1.64 (0.88, 3.06) | 1.70 (0.87, 3.32) | 41 | 1.74 (0.89, 3.37) | 1.62 (0.79, 3.31) |
| Q4 | 137 | 30 | 19 | 1.69 (0.74, 3.86) | 1.24 (0.48, 3.20) | 64 | 1.78 (0.97, 3.25) | 1.84 (0.95, 3.55) | 24 | 0.91 (0.45, 1.85) | 0.83 (0.38, 1.80) |
| Quartiles |  |  |  |  |  |  |  |  |  |  |  |
| Q1 | 55 | 40 | 15 | Ref | Ref | 48 | Ref | Ref | 35 | Ref | Ref |
| Q2-Q4 | 138 | 85 | 53 | 1.66 (0.84, 3.30) | 1.34 (0.61, 2.96) | 159 | 1.56 (0.95, 2.56) | 1.63 (0.95, 2.80) | 115 | 1.55 (0.91, 2.64) | 1.53 (0.86, 2.74) |

^1^- Adjusted for maternal age, maternal education, parity, smoking status, diabetes, BMI, race/ethnicity (black, white, Hispanic, others), preterm status and year of birth

ASD: Autism spectrum disorder; ADHD: Attention-deficit/hyperactivity disorder; DD: Developmental disabilities; 5-MTX: 5-methoxytryptophol

## eTable 3: Association between cord 5-methoxytryptophol (5-MTX) and ASD, ADHD, and other DD – Only in girls

|  |  |  | ASD | | | ADHD | | | Other DD | | |
| --- | --- | --- | --- | --- | --- | --- | --- | --- | --- | --- | --- |
|  | Total n | ND n | n | Crude | Adjusted^1^ | n | Crude | Adjusted^1^ | n | Crude | Adjusted^1^ |
| **5-MTX** |  |  |  |  |  |  |  |  |  |  |  |
| Continuous | 446 | 201 | 19 | 0.28 (0.15, 0.54) | 0.37 (0.18, 0.77) | 62 | 0.58 (0.42, 0.80) | 0.56 (0.39, 0.81) | 164 | 0.96 (0.76, 1.21) | 1.00 (0.77, 1.28) |
| Quartiles |  |  |  |  |  |  |  |  |  |  |  |
| Q1 | 112 | 43 | 11 | Ref | Ref | 20 | Ref | Ref | 38 | Ref | Ref |
| Q2 | 111 | 49 | 4 | 0.32 (0.09, 1.08) | 0.56 (0.14, 2.32) | 16 | 0.70 (0.32, 1.52) | 0.54 (0.22, 1.30) | 42 | 0.97 (0.53, 1.77) | 1.12 (0.58, 2.15) |
| Q3 | 112 | 54 | 2 | 0.14 (0.03, 0.69) | 0.21 (0.04, 1.21) | 14 | 0.56 (0.25, 1.23) | 0.49 (0.20, 1.17) | 42 | 0.88 (0.49, 1.59) | 1.16 (0.61, 2.24) |
| Q4 | 111 | 55 | 2 | 0.14 (0.03, 0.68) | 0.30 (0.05, 1.72) | 12 | 0.47 (0.21, 1.06) | 0.42 (0.17, 1.09) | 42 | 0.86 (0.48, 1.56) | 0.98 (0.52, 1.87) |
| Quartiles |  |  |  |  |  |  |  |  |  |  |  |
| Q1 | 112 | 43 | 11 | Ref | Ref | 20 | Ref | Ref | 38 | Ref | Ref |
| Q2-Q4 | 334 | 158 | 8 | 0.20 (0.08, 0.51) | 0.36 (0.12, 1.10) | 42 | 0.56 (0.30, 1.03) | 0.52 (0.26, 1.03) | 126 | 0.88 (0.52, 1.47) | 1.10 (0.62, 1.95) |
| **Tryptophan** |  |  |  |  |  |  |  |  |  |  |  |
| Continuous | 446 | 201 | 19 | 0.86 (0.55, 1.34) | 0.68 (0.36, 1.29) | 62 | 1.25 (0.95, 1.66) | 1.19 (0.88, 1.62) | 164 | 1.00 (0.83, 1.21) | 0.95 (0.78, 1.17) |
| Quartiles |  |  |  |  |  |  |  |  |  |  |  |
| Q1 | 112 | 52 | 6 | Ref | Ref | 10 | Ref | Ref | 44 | Ref | Ref |
| Q2 | 111 | 54 | 4 | 0.64 (0.17, 2.41) | 0.46 (0.10, 2.12) | 16 | 1.54 (0.64, 3.70) | 1.96 (0.74, 5.15) | 37 | 0.81 (0.45, 1.45) | 0.62 (0.33, 1.19) |
| Q3 | 112 | 42 | 9 | 1.86 (0.61, 5.63) | 0.76 (0.18, 3.13) | 18 | 2.23 (0.93, 5.34) | 2.49 (0.94, 6.59) | 43 | 1.21 (0.67, 2.17) | 0.84 (0.44, 1.59) |
| Q4 | 111 | 53 | 0 | Not estimable | Not estimable | 18 | 1.77 (0.75, 4.18) | 1.58 (0.60, 4.12) | 40 | 0.89 (0.50, 1.58) | 0.79 (0.43, 1.47) |
| Quartiles |  |  |  |  |  |  |  |  |  |  |  |
| Q1 | 112 | 52 | 6 | Ref | Ref | 10 | Ref | Ref | 44 | Ref | Ref |
| Q2-Q4 | 334 | 149 | 13 | 0.76 (0.27, 2.09) | 0.41 (0.12, 1.44) | 52 | 1.81 (0.86, 3.83) | 1.95 (0.86, 4.44) | 120 | 0.95 (0.60, 1.52) | 0.75 (0.45, 1.25) |
| **5-** **hydroxytryptophan** |  |  |  |  |  |  |  |  |  |  |  |
| Continuous | 446 | 201 | 19 | 1.31 (0.78, 2.21) | 1.00 (0.54, 1.83) | 62 | 1.39 (1.02, 1.89) | 1.31 (0.93, 1.85) | 164 | 1.03 (0.83, 1.29) | 0.95 (0.75, 1.21) |
| Quartiles |  |  |  |  |  |  |  |  |  |  |  |
| Q1 | 113 | 55 | 1 | Ref | Ref | 9 | Ref | Ref | 48 | Ref | Ref |
| Q2 | 110 | 49 | 7 | 7.86 (0.93, 66.14) | 15.29 (1.41, 165.77) | 19 | 2.37 (0.98, 5.72) | 2.00 (0.77, 5.20) | 35 | 0.82 (0.46, 1.46) | 0.89 (0.47, 1.66) |
| Q3 | 112 | 49 | 8 | 8.98 (1.08, 74.38) | 11.02 (1.07, 113.89) | 14 | 1.75 (0.69, 4.39) | 1.53 (0.57, 4.14) | 41 | 0.96 (0.54, 1.69) | 0.82 (0.45, 1.52) |
| Q4 | 111 | 48 | 3 | 3.44 (0.35, 34.15) | 1.75 (0.13, 23.70) | 20 | 2.55 (1.06, 6.12) | 2.17 (0.84, 5.58) | 40 | 0.95 (0.54, 1.69) | 0.79 (0.43, 1.47) |
| Quartiles |  |  |  |  |  |  |  |  |  |  |  |
| Q1 | 113 | 55 | 1 | Ref | Ref | 9 | Ref | Ref | 48 | Ref | Ref |
| Q2-Q4 | 333 | 146 | 18 | 6.78 (0.88, 52.01) | 7.71 (0.89, 66.73) | 53 | 2.22 (1.03, 4.80) | 1.90 (0.83, 4.33) | 116 | 0.91 (0.58, 1.44) | 0.83 (0.51, 1.36) |
| **Serotonin** |  |  |  |  |  |  |  |  |  |  |  |
| Continuous | 446 | 201 | 19 | 1.14 (0.69, 1.89) | 0.90 (0.49, 1.62) | 62 | 1.00 (0.74, 1.36) | 1.09 (0.78, 1.51) | 164 | 1.07 (0.87, 1.33) | 1.04 (0.83, 1.31) |
| Quartiles |  |  |  |  |  |  |  |  |  |  |  |
| Q1 | 112 | 55 | 3 | Ref | Ref | 11 | Ref | Ref | 43 | Ref | Ref |
| Q2 | 111 | 44 | 9 | 3.75 (0.96, 14.69) | 4.04 (0.78, 20.98) | 18 | 2.05 (0.88, 4.78) | 2.79 (1.07, 7.28) | 40 | 1.16 (0.65, 2.09) | 1.31 (0.69, 2.48) |
| Q3 | 112 | 57 | 3 | 0.96 (0.19, 4.99) | 0.85 (0.12, 5.83) | 21 | 1.84 (0.81, 4.17) | 2.83 (1.13, 7.11) | 31 | 0.70 (0.38, 1.26) | 0.75 (0.39, 1.43) |
| Q4 | 111 | 45 | 4 | 1.63 (0.35, 7.66) | 0.90 (0.15, 5.49) | 12 | 1.33 (0.54, 3.31) | 1.83 (0.67, 4.99) | 50 | 1.42 (0.81, 2.51) | 1.35 (0.72, 2.52) |
| Quartiles |  |  |  |  |  |  |  |  |  |  |  |
| Q1 | 112 | 55 | 3 | Ref | Ref | 11 | Ref | Ref | 43 | Ref | Ref |
| Q2-Q4 | 334 | 146 | 16 | 2.01 (0.56, 7.17) | 1.63 (0.38, 7.11) | 51 | 1.75 (0.85, 3.59) | 2.49 (1.11, 5.57) | 121 | 1.06 (0.67, 1.69) | 1.11 (0.66, 1.85) |
| **N-acetyltryptophan** |  |  |  |  |  |  |  |  |  |  |  |
| Continuous | 446 | 201 | 19 | 1.47 (0.90, 2.40) | 1.30 (0.75, 2.19) | 62 | 1.35 (1.00, 1.82) | 1.26 (0.90, 1.76) | 164 | 1.11 (0.90, 1.36) | 0.98 (0.79, 1.23) |
| Quartiles |  |  |  |  |  |  |  |  |  |  |  |
| Q1 | 112 | 60 | 2 | Ref | Ref | 8 | Ref | Ref | 42 | Ref | Ref |
| Q2 | 111 | 49 | 4 | 2.45 (0.43, 13.94) | 2.96 (0.41, 21.53) | 17 | 2.60 (1.04, 6.54) | 1.87 (0.70, 5.00) | 41 | 1.20 (0.67, 2.12) | 0.96 (0.52, 1.77) |
| Q3 | 112 | 45 | 7 | 4.67 (0.93, 23.54) | 4.72 (0.78, 28.51) | 21 | 3.50 (1.42, 8.62) | 2.83 (1.08, 7.44) | 39 | 1.24 (0.69, 2.22) | 1.00 (0.53, 1.88) |
| Q4 | 111 | 47 | 6 | 3.83 (0.74, 19.85) | 3.58 (0.57, 22.59) | 16 | 2.55 (1.01, 6.48) | 2.05 (0.75, 5.56) | 42 | 1.28 (0.72, 2.26) | 0.96 (0.51, 1.80) |
| Quartiles |  |  |  |  |  |  |  |  |  |  |  |
| Q1 | 112 | 60 | 2 | Ref | Ref | 8 | Ref | Ref | 42 | Ref | Ref |
| Q2-Q4 | 334 | 141 | 17 | 3.62 (0.81, 16.15) | 3.86 (0.74, 20.03) | 54 | 2.87 (1.29, 6.40) | 2.23 (0.96, 5.20) | 122 | 1.24 (0.78, 1.96) | 0.97 (0.59, 1.60) |

^1^- Adjusted for maternal age, maternal education, parity, smoking status, diabetes, BMI, race/ethnicity (black, white, Hispanic, others), preterm status and year of birth

ASD: Autism spectrum disorder; ADHD: Attention-deficit/hyperactivity disorder; DD: Developmental disabilities; 5-MTX: 5-methoxytryptophol

## eTable 4: Association between cord 5-methoxytryptophol (5-MTX) and ASD, ADHD, and other DD – Only in Blacks

|  |  |  | ASD | | | ADHD | | | Other DD | | |
| --- | --- | --- | --- | --- | --- | --- | --- | --- | --- | --- | --- |
|  | Total n | ND n | n | Crude | Adjusted^1^ | n | Crude | Adjusted^1^ | n | Crude | Adjusted^1^ |
| **5-MTX** |  |  |  |  |  |  |  |  |  |  |  |
| Continuous | 633 | 222 | 43 | 0.50 (0.34, 0.73) | 0.61 (0.40, 0.91) | 170 | 0.80 (0.65, 0.98) | 0.85 (0.66, 1.08) | 198 | 1.01 (0.81, 1.25) | 1.03 (0.82, 1.30) |
| Quartiles |  |  |  |  |  |  |  |  |  |  |  |
| Q1 | 160 | 45 | 23 | Ref | Ref | 51 | Ref | Ref | 41 | Ref | Ref |
| Q2 | 157 | 63 | 6 | 0.19 (0.07, 0.49) | 0.16 (0.05, 0.50) | 40 | 0.56 (0.32, 0.98) | 0.54 (0.28, 1.04) | 48 | 0.84 (0.47, 1.47) | 0.87 (0.47, 1.58) |
| Q3 | 158 | 56 | 5 | 0.17 (0.06, 0.50) | 0.26 (0.07, 0.87) | 34 | 0.54 (0.30, 0.96) | 0.51 (0.26, 1.02) | 63 | 1.23 (0.71, 2.15) | 1.48 (0.81, 2.69) |
| Q4 | 158 | 58 | 9 | 0.30 (0.13, 0.72) | 0.42 (0.15, 1.16) | 45 | 0.68 (0.39, 1.20) | 0.78 (0.40, 1.50) | 46 | 0.87 (0.49, 1.54) | 0.87 (0.48, 1.61) |
| Quartiles |  |  |  |  |  |  |  |  |  |  |  |
| Q1 | 160 | 45 | 23 | Ref | Ref | 51 | Ref | Ref | 41 | Ref | Ref |
| Q2-Q4 | 473 | 177 | 20 | 0.22 (0.11, 0.44) | 0.27 (0.12, 0.60) | 119 | 0.59 (0.37, 0.94) | 0.60 (0.35, 1.04) | 157 | 0.97 (0.61, 1.56) | 1.04 (0.63, 1.73) |
| **Tryptophan** |  |  |  |  |  |  |  |  |  |  |  |
| Continuous | 633 | 222 | 43 | 0.88 (0.63, 1.21) | 0.79 (0.52, 1.21) | 170 | 1.18 (0.96, 1.44) | 1.23 (0.97, 1.55) | 198 | 0.93 (0.76, 1.13) | 0.93 (0.75, 1.14) |
| Quartiles |  |  |  |  |  |  |  |  |  |  |  |
| Q1 | 159 | 52 | 15 | Ref | Ref | 36 | Ref | Ref | 56 | Ref | Ref |
| Q2 | 158 | 65 | 6 | 0.32 (0.12, 0.88) | 0.19 (0.06, 0.63) | 39 | 0.87 (0.48, 1.55) | 0.98 (0.50, 1.90) | 48 | 0.69 (0.40, 1.17) | 0.62 (0.36, 1.10) |
| Q3 | 158 | 53 | 14 | 0.92 (0.40, 2.08) | 0.46 (0.17, 1.26) | 45 | 1.23 (0.69, 2.19) | 1.40 (0.71, 2.77) | 46 | 0.81 (0.47, 1.39) | 0.73 (0.40, 1.32) |
| Q4 | 158 | 52 | 8 | 0.53 (0.21, 1.37) | 0.44 (0.14, 1.38) | 50 | 1.39 (0.78, 2.47) | 1.52 (0.78, 2.99) | 48 | 0.86 (0.50, 1.48) | 0.86 (0.48, 1.52) |
| Quartiles |  |  |  |  |  |  |  |  |  |  |  |
| Q1 | 159 | 52 | 15 | Ref | Ref | 36 | Ref | Ref | 56 | Ref | Ref |
| Q2-Q4 | 474 | 170 | 28 | 0.57 (0.28, 1.15) | 0.35 (0.15, 0.83) | 134 | 1.14 (0.70, 1.84) | 1.27 (0.73, 2.22) | 142 | 0.78 (0.50, 1.20) | 0.73 (0.46, 1.16) |
| **5-** **hydroxytryptophan** |  |  |  |  |  |  |  |  |  |  |  |
| Continuous | 633 | 222 | 43 | 1.31 (0.92, 1.86) | 1.38 (0.92, 2.07) | 170 | 1.39 (1.12, 1.72) | 1.46 (1.14, 1.88) | 198 | 0.95 (0.77, 1.17) | 0.88 (0.70, 1.10) |
| Quartiles |  |  |  |  |  |  |  |  |  |  |  |
| Q1 | 160 | 62 | 8 | Ref | Ref | 29 | Ref | Ref | 61 | Ref | Ref |
| Q2 | 157 | 53 | 13 | 1.90 (0.73, 4.93) | 2.76 (0.89, 8.53) | 45 | 1.82 (1.00, 3.29) | 2.60 (1.30, 5.19) | 46 | 0.88 (0.52, 1.50) | 0.89 (0.51, 1.56) |
| Q3 | 158 | 63 | 12 | 1.48 (0.56, 3.86) | 2.12 (0.69, 6.48) | 40 | 1.36 (0.75, 2.46) | 1.55 (0.78, 3.05) | 43 | 0.69 (0.41, 1.17) | 0.66 (0.38, 1.16) |
| Q4 | 158 | 44 | 10 | 1.76 (0.64, 4.82) | 2.10 (0.63, 7.04) | 56 | 2.72 (1.51, 4.92) | 3.29 (1.62, 6.66) | 48 | 1.11 (0.65, 1.90) | 0.90 (0.50, 1.63) |
| Quartiles |  |  |  |  |  |  |  |  |  |  |  |
| Q1 | 160 | 62 | 8 | Ref | Ref | 29 | Ref | Ref | 61 | Ref | Ref |
| Q2-Q4 | 473 | 160 | 35 | 1.70 (0.75, 3.86) | 2.31 (0.88, 6.06) | 141 | 1.88 (1.15, 3.09) | 2.32 (1.31, 4.12) | 137 | 0.87 (0.57, 1.33) | 0.80 (0.51, 1.26) |
| **Serotonin** |  |  |  |  |  |  |  |  |  |  |  |
| Continuous | 633 | 222 | 43 | 1.05 (0.75, 1.49) | 1.02 (0.68, 1.53) | 170 | 1.06 (0.87, 1.30) | 1.09 (0.86, 1.37) | 198 | 0.91 (0.74, 1.10) | 0.88 (0.71, 1.09) |
| Quartiles |  |  |  |  |  |  |  |  |  |  |  |
| Q1 | 159 | 51 | 9 | Ref | Ref | 40 | Ref | Ref | 59 | Ref | Ref |
| Q2 | 158 | 55 | 12 | 1.24 (0.48, 3.18) | 0.70 (0.22, 2.20) | 42 | 0.97 (0.55, 1.73) | 0.78 (0.39, 1.55) | 49 | 0.77 (0.45, 1.32) | 0.65 (0.36, 1.17) |
| Q3 | 158 | 68 | 11 | 0.92 (0.35, 2.38) | 0.78 (0.25, 2.47) | 37 | 0.69 (0.39, 1.23) | 0.72 (0.37, 1.42) | 42 | 0.53 (0.31, 0.91) | 0.51 (0.29, 0.92) |
| Q4 | 158 | 48 | 11 | 1.30 (0.49, 3.41) | 1.00 (0.32, 3.15) | 51 | 1.35 (0.76, 2.40) | 1.42 (0.73, 2.77) | 48 | 0.86 (0.50, 1.50) | 0.79 (0.44, 1.44) |
| Quartiles |  |  |  |  |  |  |  |  |  |  |  |
| Q1 | 159 | 51 | 9 | Ref | Ref | 40 | Ref | Ref | 59 | Ref | Ref |
| Q2-Q4 | 474 | 171 | 34 | 1.13 (0.51, 2.50) | 0.82 (0.31, 2.15) | 170 | 0.97 (0.60, 1.55) | 0.94 (0.54, 1.64) | 139 | 0.70 (0.45, 1.09) | 0.64 (0.40, 1.02) |
| **N-acetyltryptophan** |  |  |  |  |  |  |  |  |  |  |  |
| Continuous | 633 | 222 | 43 | 1.22 (0.89, 1.68) | 1.04 (0.72, 1.50) | 170 | 1.31 (1.08, 1.59) | 1.29 (1.03, 1.62) | 198 | 1.11 (0.91, 1.34) | 1.03 (0.83, 1.27) |
| Quartiles |  |  |  |  |  |  |  |  |  |  |  |
| Q1 | 159 | 63 | 10 | Ref | Ref | 39 | Ref | Ref | 47 | Ref | Ref |
| Q2 | 158 | 60 | 11 | 1.16 (0.46, 2.92) | 0.85 (0.28, 2.58) | 36 | 0.97 (0.55, 1.72) | 1.03 (0.53, 2.01) | 51 | 1.14 (0.67, 1.94) | 0.91 (0.51, 1.61) |
| Q3 | 158 | 50 | 8 | 1.01 (0.37, 2.74) | 1.10 (0.36, 3.34) | 45 | 1.45 (0.82, 2.56) | 1.61 (0.83, 3.12) | 55 | 1.47 (0.86, 2.52) | 1.31 (0.74, 2.32) |
| Q4 | 158 | 49 | 14 | 1.80 (0.74, 4.40) | 1.18 (0.41, 3.43) | 50 | 1.65 (0.94, 2.89) | 1.59 (0.82, 3.09) | 45 | 1.23 (0.71, 2.14) | 0.97 (0.53, 1.78) |
| Quartiles |  |  |  |  |  |  |  |  |  |  |  |
| Q1 | 159 | 63 | 10 | Ref | Ref | 39 | Ref | Ref | 47 | Ref | Ref |
| Q2-Q4 | 474 | 159 | 33 | 1.31 (0.61, 2.81) | 1.04 (0.43, 2.50) | 131 | 1.33 (0.84, 2.11) | 1.39 (0.80, 2.39) | 151 | 1.27 (0.82, 1.97) | 1.06 (0.66, 1.70) |

1. Adjusted for maternal age, maternal education, parity, smoking status, diabetes, BMI, sex, preterm status and year of birth

ASD: Autism spectrum disorder; ADHD: Attention-deficit/hyperactivity disorder; DD: Developmental disabilities; 5-MTX: 5-methoxytryptophol

## eTable 5: Association between cord 5-methoxytryptophol (5-MTX) and ASD, ADHD, and other DD – Only in non-blacks

|  |  |  | ASD | | | ADHD | | | Other DD | | |
| --- | --- | --- | --- | --- | --- | --- | --- | --- | --- | --- | --- |
|  | Total n | ND n | n | Crude | Adjusted^1^ | n | Crude | Adjusted^1^ | n | Crude | Adjusted^1^ |
| **5-MTX** |  |  |  |  |  |  |  |  |  |  |  |
| Continuous | 363 | 104 | 44 | 0.48 (0.31, 0.73) | 0.46 (0.28, 0.77) | 99 | 0.73 (0.55, 0.97) | 0.67 (0.48, 0.95) | 116 | 1.12 (0.84, 1.50) | 1.18 (0.85, 1.63) |
| Quartiles |  |  |  |  |  |  |  |  |  |  |  |
| Q1 | 91 | 17 | 21 | Ref | Ref | 36 | Ref | Ref | 17 | Ref | Ref |
| Q2 | 91 | 29 | 6 | 0.17 (0.06, 0.50) | 0.22 (0.06, 0.79) | 24 | 0.39 (0.18, 0.86) | 0.44 (0.17, 1.17) | 32 | 1.10 (0.48, 2.55) | 1.13 (0.45, 2.86) |
| Q3 | 91 | 37 | 9 | 0.20 (0.07, 0.52) | 0.29 (0.09, 0.91) | 13 | 0.17 (0.07, 0.39) | 0.18 (0.06, 0.50) | 32 | 0.86 (0.38, 1.97) | 1.05 (0.43, 2.58) |
| Q4 | 90 | 21 | 8 | 0.31 (0.11, 0.87) | 0.31 (0.08, 1.12) | 26 | 0.58 (0.26, 1.32) | 0.60 (0.22, 1.62) | 35 | 1.67 (0.70, 3.95) | 2.06 (0.78, 5.42) |
| Quartiles |  |  |  |  |  |  |  |  |  |  |  |
| Q1 | 91 | 17 | 21 | Ref | Ref | 36 | Ref | Ref | 17 | Ref | Ref |
| Q2-Q4 | 272 | 87 | 23 | 0.21 (0.10, 0.47) | 0.27 (0.10, 0.71) | 63 | 0.34 (0.18, 0.66) | 0.36 (0.16, 0.82) | 99 | 1.14 (0.55, 2.36) | 1.28 (0.56, 2.89) |
| **Tryptophan** |  |  |  |  |  |  |  |  |  |  |  |
| Continuous | 363 | 104 | 44 | 1.01 (0.71, 1.44) | 1.10 (0.71, 1.70) | 99 | 1.29 (0.98, 1.70) | 1.28 (0.93, 1.78) | 116 | 1.04 (0.81, 1.35) | 1.05 (0.80, 1.38) |
| Quartiles |  |  |  |  |  |  |  |  |  |  |  |
| Q1 | 91 | 34 | 12 | Ref | Ref | 16 | Ref | Ref | 29 | Ref | Ref |
| Q2 | 91 | 23 | 8 | 0.99 (0.35, 2.79) | 1.04 (0.29, 3.79) | 27 | 2.49 (1.11, 5.63) | 2.62 (0.99, 6.96) | 33 | 1.68 (0.81, 3.48) | 1.81 (0.82, 3.98) |
| Q3 | 91 | 20 | 17 | 2.41 (0.96, 6.06) | 3.21 (0.99, 10.36) | 29 | 3.08 (1.35, 7.02) | 2.77 (1.04, 7.36) | 25 | 1.47 (0.68, 3.16) | 1.47 (0.65, 3.35) |
| Q4 | 90 | 27 | 7 | 0.73 (0.25, 2.12) | 1.05 (0.30, 3.69) | 27 | 2.13 (0.96, 4.72) | 2.17 (0.85, 5.53) | 29 | 1.26 (0.61, 2.59) | 1.34 (0.61, 2.94) |
| Quartiles |  |  |  |  |  |  |  |  |  |  |  |
| Q1 | 91 | 34 | 12 | Ref | Ref | 16 | Ref | Ref | 29 | Ref | Ref |
| Q2-Q4 | 272 | 70 | 32 | 1.30 (0.59, 2.82) | 1.63 (0.61, 4.40) | 83 | 2.52 (1.28, 4.94) | 2.48 (1.13, 5.47) | 87 | 1.46 (0.81, 2.62) | 1.53 (0.81, 2.88) |
| **5-** **hydroxytryptophan** |  |  |  |  |  |  |  |  |  |  |  |
| Continuous | 363 | 104 | 44 | 1.24 (0.85, 1.81) | 1.37 (0.86, 2.20) | 99 | 1.32 (0.98, 1.77) | 1.19 (0.84, 1.68) | 116 | 1.13 (0.86, 1.50) | 1.14 (0.84, 1.55) |
| Quartiles |  |  |  |  |  |  |  |  |  |  |  |
| Q1 | 92 | 35 | 10 | Ref | Ref | 20 | Ref | Ref | 27 | Ref | Ref |
| Q2 | 90 | 33 | 11 | 1.75 (0.64, 4.80) | 1.62 (0.47, 5.59) | 23 | 1.83 (0.82, 4.08) | 1.37 (0.51, 3.68) | 34 | 2.00 (0.96, 4.18) | 1.77 (0.81, 3.85) |
| Q3 | 91 | 32 | 13 | 2.17 (0.81, 5.81) | 1.84 (0.55, 6.23) | 26 | 2.17 (0.98, 4.80) | 2.28 (0.90, 5.77) | 31 | 1.91 (0.91, 4.04) | 1.80 (0.81, 3.99) |
| Q4 | 90 | 36 | 10 | 1.35 (0.49, 3.71) | 1.61 (0.46, 5.63) | 30 | 2.02 (0.94, 4.32) | 1.51 (0.60, 3.79) | 24 | 1.20 (0.57, 2.53) | 1.24 (0.55, 2.79) |
| Quartiles |  |  |  |  |  |  |  |  |  |  |  |
| Q1 | 92 | 35 | 10 | Ref | Ref | 20 | Ref | Ref | 27 | Ref | Ref |
| Q2-Q4 | 271 | 69 | 34 | 1.72 (0.76, 3.89) | 1.69 (0.61, 4.68) | 79 | 2.00 (1.06, 3.79) | 1.71 (0.80, 3.66) | 89 | 1.67 (0.92, 3.02) | 1.60 (0.84, 3.02) |
| **Serotonin** |  |  |  |  |  |  |  |  |  |  |  |
| Continuous | 363 | 104 | 44 | 1.31 (0.93, 1.86) | 1.45 (0.94, 2.25) | 99 | 0.99 (0.74, 1.32) | 1.04 (0.72, 1.48) | 116 | 0.88 (0.67, 1.15) | 0.92 (0.68, 1.25) |
| Quartiles |  |  |  |  |  |  |  |  |  |  |  |
| Q1 | 91 | 24 | 7 | Ref | Ref | 22 | Ref | Ref | 38 | Ref | Ref |
| Q2 | 91 | 32 | 10 | 1.07 (0.36, 3.22) | 0.98 (0.24, 3.97) | 19 | 0.65 (0.29, 1.46) | 0.90 (0.34, 2.38) | 30 | 0.59 (0.29, 1.21) | 0.59 (0.27, 1.30) |
| Q3 | 91 | 21 | 13 | 2.12 (0.71, 6.31) | 2.58 (0.69, 9.69) | 37 | 1.92 (0.87, 4.23) | 2.27 (0.87, 5.93) | 20 | 0.60 (0.27, 1.34) | 0.65 (0.28, 1.52) |
| Q4 | 90 | 27 | 14 | 1.78 (0.62, 5.14) | 1.98 (0.53, 7.43) | 21 | 0.85 (0.38, 1.91) | 0.93 (0.35, 2.48) | 28 | 0.65 (0.31, 1.37) | 0.72 (0.32, 1.63) |
| Quartiles |  |  |  |  |  |  |  |  |  |  |  |
| Q1 | 91 | 24 | 7 | Ref | Ref | 22 | Ref | Ref | 38 | Ref | Ref |
| Q2-Q4 | 272 | 80 | 37 | 1.59 (0.63, 4.01) | 1.74 (0.56, 5.48) | 77 | 1.05 (0.54, 2.03) | 1.25 (0.56, 2.78) | 78 | 0.62 (0.34, 1.12) | 0.65 (0.34, 1.25) |
| **N-acetyltryptophan** |  |  |  |  |  |  |  |  |  |  |  |
| Continuous | 363 | 104 | 44 | 1.28(0.89, 1.83) | 1.14 (0.75, 1.72) | 99 | 1.45 (1.08, 1.94) | 1.33 (0.94, 1.89) | 116 | 1.01 (0.76, 1.35) | 0.92 (0.67, 1.25) |
| Quartiles |  |  |  |  |  |  |  |  |  |  |  |
| Q1 | 91 | 36 | 8 | Ref | Ref | 17 | Ref | Ref | 30 | Ref | Ref |
| Q2 | 91 | 23 | 9 | 1.76 (0.59, 5.22) | 2.04 (0.54, 7.67) | 22 | 2.03 (0.89, 4.60) | 2.48 (0.94, 6.53) | 37 | 1.93 (0.95, 3.93) | 2.22 (1.03, 4.77) |
| Q3 | 91 | 20 | 14 | 3.15 (1.13, 8.79) | 3.44 (0.99, 12.01) | 28 | 2.96 (1.31, 6.69) | 3.32 (1.20, 9.24) | 29 | 1.74 (0.82, 3.68) | 1.48 (0.65, 3.37) |
| Q4 | 90 | 25 | 13 | 2.34 (0.85, 6.48) | 1.65 (0.53, 5.20) | 32 | 2.71 (1.24, 5.91) | 2.24 (0.87, 5.79) | 20 | 0.96 (0.45, 2.06) | 0.82 (0.36, 1.88) |
| Quartiles |  |  |  |  |  |  |  |  |  |  |  |
| Q1 | 91 | 36 | 8 | Ref | Ref | 17 | Ref | Ref | 30 | Ref | Ref |
| Q2-Q4 | 272 | 68 | 36 | 2.38 (1.00, 5.66) | 2.15 (0.80, 5.78) | 82 | 2.55 (1.32, 4.94) | 2.58 (1.15, 5.79) | 86 | 1.52 (0.85, 2.71) | 1.47 (0.78, 2.76) |

^1^Adjusted for maternal age, maternal education, parity, smoking status, diabetes, BMI, sex, preterm status and year of birth

ASD: Autism spectrum disorder; ADHD: Attention-deficit/hyperactivity disorder; DD: Developmental disabilities; 5-MTX: 5-methoxytryptophol

## eTable 6: Association between cord 5-methoxytryptophol (5-MTX) and ASD, ADHD, and other DD, stratified by term and preterm births

|  |  |  | ASD | | | ADHD | | | Other DD | | |
| --- | --- | --- | --- | --- | --- | --- | --- | --- | --- | --- | --- |
|  | Total n | ND n | n | Crude | Adjusted^1^ | n | Crude | Adjusted^1^ | n | Crude | Adjusted^1^ |
| **Term children** | | | | | | | | | | | |
| **5-methoxytryptophol** | | | | | | | | | | | |
| Continuous | 819 | 299 | 62 | 0.54 (0.39, 0.74) | 0.55 (0.39, 0.79) | 212 | 0.74 (0.61, 0.89) | 0.74 (0.60, 0.91) | 246 | 0.99 (0.81, 1.20) | 1.05 (0.85, 1.29) |
| Quartiles |  |  |  |  |  |  |  |  |  |  |  |
| Q1 | 205 | 59 | 29 | Ref | Ref | 68 | Ref | Ref | 49 | Ref | Ref |
| Q2 | 205 | 84 | 8 | 0.19 (0.08, 0.45) | 0.21 (0.08, 0.54) | 53 | 0.55 (0.34, 0.89) | 0.52 (0.30, 0.91) | 60 | 0.86 (0.52, 1.42) | 0.88 (0.52, 1.50) |
| Q3 | 205 | 81 | 11 | 0.28 (0.13, 0.60) | 0.34 (0.14, 0.83) | 37 | 0.40 (0.24, 0.67) | 0.38 (0.21, 0.69) | 76 | 1.13 (0.69, 1.85) | 1.34 (0.79, 2.27) |
| Q4 | 204 | 75 | 14 | 0.38 (0.18, 0.78) | 0.38 (0.16, 0.88) | 54 | 0.62 (0.38, 1.02) | 0.62 (0.35, 1.09) | 61 | 0.98 (0.59, 1.63) | 1.11 (0.65, 1.89) |
| Quartiles |  |  |  |  |  |  |  |  |  |  |  |
| Q1 | 205 | 59 | 29 | Ref | Ref | 68 | Ref | Ref | 49 | Ref | Ref |
| Q2-Q4 | 614 | 240 | 33 | 0.28 (0.16, 0.50) | 0.31 (0.16, 0.60) | 144 | 0.52 (0.35, 0.78) | 0.50 (0.32, 0.80) | 197 | 0.99 (0.65, 1.51) | 1.10 (0.70, 1.72) |
| **Tryptophan** |  |  |  |  |  |  |  |  |  |  |  |
| Continuous | 819 | 299 | 62 | 0.95 (0.73, 1.25) | 0.95 (0.69, 1.32) | 212 | 1.21 (1.01, 1.44) | 1.22 (1.00, 1.49) | 246 | 0.92 (0.78,1.09) | 0.94 (0.79, 1.12) |
| Quartiles |  |  |  |  |  |  |  |  |  |  |  |
| Q1 | 205 | 77 | 17 | Ref | Ref | 38 | Ref | Ref | 73 | Ref | Ref |
| Q2 | 205 | 84 | 9 | 0.49 (0.20, 1.15) | 0.43 (0.17, 1.13) | 54 | 1.30 (0.78, 2.19) | 1.40 (0.79, 2.51) | 58 | 0.73 (0.46, 1.16) | 0.72 (0.44, 1.17) |
| Q3 | 205 | 59 | 26 | 2.00 (0.99, 4.02) | 1.83 (0.80, 4.15) | 59 | 2.03 (1.19, 3.44) | 2.33 (1.27, 4.28) | 61 | 1.09 (0.67, 1.76) | 1.00 (0.60, 1.66) |
| Q4 | 204 | 79 | 10 | 0.57 (0.25, 1.33) | 0.56 (0.22, 1.43) | 61 | 1.56 (0.94, 2.61) | 1.62 (0.91, 2.87) | 54 | 0.72 (0.45, 1.16) | 0.72 (0.44, 1.18) |
| Quartiles |  |  |  |  |  |  |  |  |  |  |  |
| Q1 | 205 | 77 | 17 | Ref | Ref | 38 | Ref | Ref | 73 | Ref | Ref |
| Q2-Q4 | 614 | 222 | 45 | 0.92 (0.50, 1.70) | 0.83 (0.41, 1.67) | 174 | 1.59 (1.03, 2.46) | 1.71 (1.05, 2.79) | 173 | 0.82 (0.56, 1.20) | 0.79 (0.53, 1.18) |
| **5-** **hydroxytryptophan** |  |  |  |  |  |  |  |  |  |  |  |
| Continuous | 819 | 299 | 62 | 1.39 (1.03, 1.88) | 1.46 (0.04, 2.05) | 212 | 1.30 (1.07, 1.57) | 1.27 (1.03, 1.58) | 246 | 0.92 (0.77, 1.11) | 0.91 (0.75, 1.10) |
| Quartiles |  |  |  |  |  |  |  |  |  |  |  |
| Q1 | 205 | 82 | 9 | Ref | Ref | 40 | Ref | Ref | 74 | Ref | Ref |
| Q2 | 205 | 67 | 18 | 2.45 (1.03, 5.80) | 2.43 (0.95, 6.25) | 58 | 1.77 (1.06, 2.97) | 2.14 (1.19, 3.82) | 62 | 1.03 (0.64, 1.64) | 1.02 (0.63, 1.68) |
| Q3 | 205 | 81 | 21 | 2.36 (1.02, 5.47) | 2.15 (0.85, 5.43) | 46 | 1.16 (0.69, 1.96 | 1.32 (0.74, 2.35) | 57 | 0.78 (0.49, 1.24) | 0.71 (0.44, 1.16) |
| Q4 | 204 | 69 | 14 | 1.85 (0.75, 4.53) | 1.79 (0.66, 4.85) | 68 | 2.02 (1.22, 3.35) | 2.12 (1.20, 3.75) | 53 | 0.85 (0.53, 1.37) | 0.78 (0.47, 1.29) |
| Quartiles |  |  |  |  |  |  |  |  |  |  |  |
| Q1 | 91 | 82 | 9 | Ref | Ref | 40 | Ref | Ref | 74 | Ref | Ref |
| Q2-Q4 | 270 | 217 | 53 | 2.23 (1.05, 4.72) | 2.13 (0.94, 4.83) | 172 | 1.62 (1.06, 2.49) | 1.82 (1.13, 2.93) | 172 | 0.88 (0.61, 1.27) | 0.83 (0.56, 1.22) |
| **Serotonin** |  |  |  |  |  |  |  |  |  |  |  |
| Continuous | 819 | 299 | 62 | 1.35 (1.02, 1.80) | 1.29 (0.94, 1.76) | 212 | 1.01 (0.85, 1.21) | 1.05 (0.86, 1.28) | 246 | 0.85 (0.71, 1.01) | 0.87 (0.72, 1.05) |
| Quartiles |  |  |  |  |  |  |  |  |  |  |  |
| Q1 | 206 | 67 | 10 | Ref | Ref | 49 | Ref | Ref | 80 | Ref | Ref |
| Q2 | 204 | 73 | 16 | 1.47 (0.62, 3.46) | 1.45 (0.54, 3.86) | 53 | 0.99 (0.60, 1.65) | 0.94 (0.53, 1.68) | 62 | 0.71 (0.45, 1.14) | 0.70 (0.42, 1.15) |
| Q3 | 205 | 90 | 16 | 1.19 (0.51, 2.79) | 1.51 (0.56, 4.06) | 53 | 0.81 (0.49, 1.33) | 0.93 (0.52, 1.64) | 46 | 0.43 (0.26, 0.69) | 0.45 (0.27, 0.76) |
| Q4 | 204 | 69 | 20 | 1.94 (0.85, 4.45) | 1.65 (0.63, 4.34) | 57 | 1.13 (0.68, 1.88) | 1.24 (0.70, 2.30) | 58 | 0.70 (0.44, 1.13) | 0.71 (0.42, 1.18) |
| Quartiles |  |  |  |  |  |  |  |  |  |  |  |
| Q1 | 206 | 67 | 10 | Ref | Ref | 49 | Ref | Ref | 80 | Ref | Ref |
| Q2-Q4 | 613 | 232 | 52 | 1.50 (0.72, 3.11) | 1.54 (0.66, 3.59) | 163 | 0.96 (0.63, 1.46) | 1.03 (0.64, 1.65) | 166 | 0.60 (0.41, 0.88) | 0.61 (0.40, 0.92) |
| **N-acetyltryptophan** |  |  |  |  |  |  |  |  |  |  |  |
| Continuous | 819 | 299 | 62 | 1.13 (0.86, 1.48) | 1.08 (0.80, 1.46) | 212 | 1.28 (1.07, 1.52) | 1.25 (1.03, 1.52) | 246 | 0.95 (0.79, 1.13) | 0.94 (0.78, 1.13) |
| Quartiles |  |  |  |  |  |  |  |  |  |  |  |
| Q1 | 205 | 83 | 13 | Ref | Ref | 42 | Ref | Ref | 67 | Ref | Ref |
| Q2 | 205 | 76 | 14 | 1.18 (0.52, 2.66) | 1.16 (0.47, 2.87) | 45 | 1.17 (0.69, 1.97) | 1.38 (0.77, 2.48) | 70 | 1.14 (0.72, 1.80) | 1.08 (0.67, 1.76) |
| Q3 | 205 | 67 | 21 | 3.00 (0.93, 4.29) | 2.29 (0.95, 5.48) | 57 | 1.68 (1.01, 2.81) | 1.89 (1.06, 3.73) | 60 | 1.11 (0.69, 1.78) | 1.07 (0.65, 1.76) |
| Q4 | 204 | 73 | 14 | 1.22 (0.54, 2.77) | 1.01 (0.40, 2.52) | 68 | 1.84 (1.12, 3.03) | 1.78 (1.01, 3.12) | 49 | 0.83 (0.51, 1.35) | 0.79 (0.47, 1.32) |
| Quartiles |  |  |  |  |  |  |  |  |  |  |  |
| Q1 | 205 | 83 | 13 | Ref | Ref | 42 | Ref | Ref | 67 | Ref | Ref |
| Q2-Q4 | 614 | 216 | 49 | 1.45 (0.75, 2.81) | 1.39 (0.67, 2.92) | 170 | 1.56 (1.02, 2.37) | 1.68 (1.04, 2.70) | 179 | 1.03 (0.70, 1.50) | 0.98 (0.66, 1.46) |
| **Only in preterm children** | | | | | | | | | | | |
| **5-methoxytryptophol** |  |  |  |  |  |  |  |  |  |  |  |
| Continuous | 177 | 27 | 25 | 0.49 (0.27, 0.87) | 0.50 (0.21, 1.16) | 57 | 0.91 (0.62, 1.34) | 0.99 (0.60, 1.65) | 68 | 1.26 (0.82, 1.92) | 1.43 (0.87, 2.36) |
| Quartiles |  |  |  |  |  |  |  |  |  |  |  |
| Q1 | 45 | 5 | 13 | Ref | Ref | 18 | Ref | Ref | 9 | Ref | Ref |
| Q2 | 44 | 6 | 5 | 0.32 (0.07, 1.54) | 0.23 (0.004, 11.23) | 12 | 0.56 (0.14, 2.24) | 0.12 (0.01, 1.19) | 21 | 1.94 (0.47, 8.05) | 2.44 (0.50, 12.03) |
| Q3 | 44 | 12 | 3 | 0.10 (0.02, 0.49) | 0.02 (0.00, 0.70) | 12 | 0.28 (0.08, 0.99) | 0.15 (0.02, 0.93) | 17 | 0.79 (0.21, 2.94) | 0.82 (0.19, 3.49) |
| Q4 | 44 | 4 | 4 | 0.38 (0.07, 2.16) | 0.56 (0.03, 10.77) | 15 | 1.04 (0.24, 4.59) | 2.07 (0.24, 17.89) | 21 | 2.92 (0.63, 13.46) | 6.27 (0.92, 42.71) |
| Quartiles |  |  |  |  |  |  |  |  |  |  |  |
| Q1 | 45 | 5 | 13 | Ref | Ref | 18 | Ref | Ref | 9 | Ref | Ref |
| Q2-Q4 | 132 | 22 | 12 | 0.21 (0.06, 0.73) | 0.11 (0.01, 1.08) | 39 | 0.49 (0.16, 1.51) | 0.35 (0.08, 1.61) | 59 | 1.49 (0.45, 4.94) | 1.65 (0.43, 6.25) |
| **Tryptophan** |  |  |  |  |  |  |  |  |  |  |  |
| Continuous | 177 | 27 | 25 | 0.80 (0.45, 1.43) | 1.07 (0.44, 2.60) | 57 | 1.19 (0.77, 1.85) | 1.89 (0.98, 3.63) | 68 | 1.14 (0.70, 1.85) | 1.30 (0.74, 2.28) |
| Quartiles |  |  |  |  |  |  |  |  |  |  |  |
| Q1 | 45 | 5 | 10 | Ref | Ref | 15 | Ref | Ref | 15 | Ref | Ref |
| Q2 | 44 | 9 | 5 | 0.28 (0.06, 1.29) | 0.51 (0.04, 7.26) | 10 | 0.37 (0.20,1.44) | 0.81 (0.13, 4.92) | 20 | 0.74 (0.21, 2.67) | 0.95 (0.19, 4.77) |
| Q3 | 44 | 8 | 6 | 0.38 (0.08, 1.69) | 0.68 (0.06, 7.67) | 14 | 0.58 (0.15, 2.21) | 0.70 (0.13, 3.81) | 16 | 0.67 (0.18, 2.50) | 0.85 (0.19, 3.84) |
| Q4 | 44 | 5 | 4 | 0.40 (0.07, 2.18) | 0.60 (0.04, 8.71) | 18 | 1.2 (0.29, 4.95) | 3.51 (0.47, 26.19) | 17 | 1.13 (0.27, 4.69) | 1.74 (0.33, 9.17) |
| Quartiles |  |  |  |  |  |  |  |  |  |  |  |
| Q1 | 45 | 5 | 10 | Ref | Ref | 15 | Ref | Ref | 15 | Ref | Ref |
| Q2-Q4 | 132 | 22 | 15 | 0.34 (0.10, 1.20) | 0.61 (0.08, 4..61) | 42 | 0.64 (0.20, 1.98) | 1.07 (0.24, 4.66) | 53 | 0.80 (0.26, 2.48) | 1.09 (0.28, 4.23) |
| **5-** **hydroxytryptophan** |  |  |  |  |  |  |  |  |  |  |  |
| Continuous | 177 | 27 | 25 | 0.95 (0.58, 1.57) | 1.15 (0.52, 2.53) | 57 | 1.41 (0.91, 2.17) | 2.62 (1.28, 5.35) | 68 | 1.21 (0.78, 1.88) | 1.41 (0.86, 2.31) |
| Quartiles |  |  |  |  |  |  |  |  |  |  |  |
| Q1 | 45 | 10 | 9 | Ref | Ref | 9 | Ref | Ref | 17 | Ref | Ref |
| Q2 | 44 | 5 | 7 | 1.56 (0.36, 6.69) | 4.94 (0.46, 53.77) | 14 | 3.11 (0.80, 12.14) | 7.25 (0.96, 54.64) | 18 | 2.12 (0.60, 7.48) | 2.96 (0.70, 12.56) |
| Q3 | 44 | 5 | 3 | 0.67 (0.12, 3.62) | 1.95 (0.09, 40.24) | 17 | 3.78 (0.99, 14.48) | 20.26 (2.10, 195.34) | 19 | 2.24 (0.64, 7.86) | 2.39 (0.56, 10.26) |
| Q4 | 44 | 7 | 6 | 0.95 (0.23, 3.92) | 3.09 (0.21, 46.21) | 17 | 2.70 (0.77, 9.51) | 24.20 (2.16, 270.69) | 14 | 1.18 (0.36, 3.90) | 1.81 (0.44, 7.44) |
| Quartiles |  |  |  |  |  |  |  |  |  |  |  |
| Q1 | 45 | 10 | 9 | Ref | Ref | 9 | Ref | Ref | 17 | Ref | Ref |
| Q2-Q4 | 132 | 17 | 16 | 1.05 (0.34, 3.24) | 3.69 (0.45, 30.02) | 48 | 3.14 (1.09, 9.03) | 12.82 (2.13, 77.28) | 51 | 1.76 (0.68, 4.58) | 2.37 (0.80, 7.02) |
| **Serotonin** |  |  |  |  |  |  |  |  |  |  |  |
| Continuous | 177 | 27 | 25 | 0.85 (0.49, 1.48) | 1.06 (0.41, 2.76) | 57 | 1.18 (0.70, 2.01) | 1.57 (0.79, 3.09) | 68 | 1.08 (0.70, 1.68) | 1.12 (0.69, 1.83) |
| Quartiles |  |  |  |  |  |  |  |  |  |  |  |
| Q1 | 45 | 8 | 7 | Ref | Ref | 11 | Ref | Ref | 19 | Ref | Ref |
| Q2 | 44 | 7 | 8 | 1.31 (0.31, 5.48) | 10.97 (0.35, 340.33) | 13 | 1.35 (0.37, 4.92) | 1.75 (0.27, 11.51) | 16 | 0.96 (0.29, 3.24) | 0.70 (0.18, 2.81) |
| Q3 | 44 | 6 | 5 | 0.95 (0.20, 4.54) | 0.49 (0.01, 15.91) | 19 | 2.30 (0.63, 8.39) | 2.42 (0.32, 18.41) | 14 | 0.98 (0.28, 3.48) | 0.71 (0.16, 3.07) |
| Q4 | 44 | 6 | 5 | 0.95 (0.20, 4.54) | 3.69 (0.10, 140.86) | 14 | 1.70 (0.45, 6.36) | 3.71 (0.53, 25.89) | 19 | 1.33 (0.39, 4.58) | 1.31 (0.33, 5.18) |
| Quartiles |  |  |  |  |  |  |  |  |  |  |  |
| Q1 | 45 | 8 | 7 | Ref | Ref | 11 | Ref | Ref | 19 | Ref | Ref |
| Q2-Q4 | 132 | 19 | 18 | 1.08 (0.33, 3.60) | 2.71 (0.18, 40.44) | 46 | 1.76 (0.61, 5.06) | 2.51 (0.49, 12.97) | 49 | 1.09 (0.41, 2.90) | 0.90 (0.29, 2.80) |
| **N-acetyltryptophan** |  |  |  |  |  |  |  |  |  |  |  |
| Continuous | 177 | 27 | 25 | 1.22 (0.73, 2.04) | 1.21 (0.59, 2.49) | 57 | 1.42 (0.89, 2.26) | 2.03 (0.97, 4.24) |  | 1.50 (0.89, 2.54) | 1.93 (1.02, 3.64) |
| Quartiles |  |  |  |  |  |  |  |  |  |  |  |
| Q1 | 45 | 10 | 8 | Ref | Ref | 13 | Ref | Ref | 14 | Ref | Ref |
| Q2 | 44 | 6 | 4 | 0.83 (0.17, 4.01) | 1.24 (0.08, 20.01) | 13 | 1.67 (0.47, 5.94) | 1.28 (0.24, 6.90) | 21 | 2.50 (0.74, 8.44) | 4.17 (0.95, 18.37) |
| Q3 | 44 | 4 | 5 | 1.56 (0.31, 7.82) | 3.58 (0.15, 86.16) | 16 | 3.08 (0.78, 12.12) | 5.47 (0.86, 34.66) | 19 | 3.39 (0.88, 13.08) | 9.96 (1.76, 56.38) |
| Q4 | 44 | 7 | 8 | 1.43 (0.36, 5.66) | 1.15 (0.14, 9.66) | 15 | 1.65 (0.49, 5.57) | 2.34 (0.43, 12.65) | 14 | 1.43 (0.42, 4.83) | 1.60 (0.40, 6.35) |
| Quartiles |  |  |  |  |  |  |  |  |  |  |  |
| Q1 | 45 | 10 | 8 | Ref | Ref | 13 | Ref | Ref | 14 | Ref | Ref |
| Q2-Q4 | 132 | 17 | 17 | 1.25 (0.40, 3.94) | 1.35 (0.21, 8.76) | 44 | 1.99 (0.74, 5.39) | 2.52 (0.66, 9.53) | 54 | 2.27 (0.85, 6.03) | 3.38 (1.09, 10.49) |

^1^- Adjusted for maternal age, maternal education, parity, smoking status, diabetes, BMI, race/ethnicity (black, white, Hispanic, others), sex and year of birth

ASD: Autism spectrum disorder; ADHD: Attention-deficit/hyperactivity disorder; DD: Developmental disabilities; 5-MTX: 5-methoxytryptophol

## eTable 7. Factors associated with cord 5-methoxytryptophol

|  | Crude | P value | Adjusted^1^ | P value |
| --- | --- | --- | --- | --- |
| **5-methoxytryptophol** | | | | |
| Maternal age (y) |  |  |  |  |
| <20 | Ref |  | Ref |  |
| ≥20 - <30 | -0.09 (-0.31, 0.14) | 0.45 | -0.02 (-0.65, 0.62) | 0.96 |
| ≥30 | -0.13 (-0.39, 0.12) | 0.31 | -0.22 (-0.90, 0.47) | 0.53 |
| Parity | -0.08 (-0.21, 0.04) | 0.20 | 0.09 (-0.17, 0.36) | 0.48 |
| Smoking | -0.004 (-0.17, 0.16) | 0.97 | -0.12 (-0.43, 0.19) | 0.45 |
| Diabetes + Obesity (BMI>=30) |  |  |  |  |
| No | Ref |  | Ref |  |
| Diabetes and/or Obesity | -0.25 (-0.57, 0.08) | 0.14 | -0.37 (-0.72, -0.02) | 0.04 |
| Preterm | -0.02 (-0.18, 0.15) | 0.85 | -0.31 (-0.62, -0.01) | 0.04 |
| Sex | -0.07 (-0.20, 0.05) | 0.24 | -0.10 (-0.34, 0.15) | 0.42 |
| Race^2^ | -0.01 (-0.14, 0.11) | 0.83 | -0.23 (-0.51, 0.04) | 0.09 |
| Year of birth | 0.01 (-0.008, 0.03) | 0.24 | 0.009 (-0.03, 0.05) | 0.65 |
| Branched chain amino acid score | 0.06 (-0.008, 0.12) | 0.08 | 0.06 (-0.06, 0.18) | 0.34 |
| Tylenol score | -0.03 (-0.10, 0.03) | 0.33 | -0.04 (-0.16, 0.09) | 0.57 |

^1^ Adjusted – Mutually adjusted for all the other covariates

^2^Blacks vs. Non-blacks

## eTable 8. Sequential modeling to identify influential covariates on the association between 5-MTX and ASD in total sample

| Sequential modeling | ASD |
| --- | --- |
| 5- MTX (Crude) | 0.49 (0.37, 0.65) |
| 5-MTX + maternal age | 0.49 (0.37, 0.65) |
| 5-MTX + maternal age + education | 0.52 (0.39, 0.69) |
| 5-MTX + maternal age + education + parity | 0.52 (0.39, 0.69) |
| 5-MTX + maternal age + education + parity + smoking | 0.53 (0.40, 0.70) |
| 5-MTX + maternal age + education + parity + smoking + diabetes | 0.53 (0.40, 0.71) |
| 5-MTX + maternal age + education + parity + smoking + diabetes + BMI | 0.53 (0.40, 0.71) |
| 5-MTX + maternal age + education + parity + smoking + diabetes + BMI + preterm | 0.55 (0.41, 0.74) |
| 5-MTX + maternal age + education + parity + smoking + diabetes + BMI + preterm + child sex | 0.56 (0.41, 0.76) |
| 5-MTX + maternal age + education + parity + smoking + diabetes + BMI + preterm + child sex + race | 0.54 (0.39, 0.74) |
| 5-MTX + maternal age + education + parity + smoking + diabetes + BMI + preterm + child sex + race + year of birth | 0.54 (0.40, 0.74) |

## eTable 9: Sensitivity Analysis: Association between 5-methoxytryptophol and ASD among those that were diagnosed at least twice, including a visit to a specialist

|  |  |  | ASD (n=53) | | |
| --- | --- | --- | --- | --- | --- |
| 5 -MTX | Total n | ND n | n | Crude  OR (95% CI) | Adjusted^1^  OR (95% CI) |
| Continuous | 379 | 326 | 53 | 0.54 (0.38, 0.76) | 0.67 (0.46, 1.00) |
| Quartiles |  |  |  |  |  |
| Q1 | 95 | 69 | 26 | Ref | Ref |
| Q2-Q4 | 284 | 257 | 27 | 0.28 (0.15, 0.51) | 0.42 (0.21, 0.87) |
| Quartiles |  |  |  |  |  |
| Q1 | 95 | 69 | 26 | Ref | Ref |
| Q2 | 96 | 89 | 7 | 0.21 (0.09, 0.51) | 0.26 (0.09, 0.75) |
| Q3 | 94 | 86 | 8 | 0.25 (0.11, 0.58) | 0.43 (0.15, 1.19) |
| Q4 | 94 | 82 | 12 | 0.39 (0.18, 0.83) | 0.61 (0.25, 1.48) |
| Tertiles |  |  |  |  |  |
| T1 | 127 | 98 | 29 | Ref | Ref |
| T2 | 126 | 118 | 8 | 0.23 (0.10, 0.52) | 0.26 (0.10, 0.70) |
| T3 | 126 | 110 | 16 | 0.49 (0.25, 0.96) | 0.83 (0.37, 1.85) |
| Quintiles |  |  |  |  |  |
| Q1 | 76 | 55 | 21 | Ref | Ref |
| Q2 | 76 | 67 | 9 | 0.35 (0.15, 0.83) | 0.36 (0.13, 1.03) |
| Q3 | 76 | 71 | 5 | 0.18 (0.07, 0.52) | 0.18 (0.05, 0.62) |
| Q4 | 76 | 67 | 9 | 0.35 (0.15, 0.83) | 0.72 (0.25, 2.07) |
| Q5 | 75 | 66 | 9 | 0.36 (0.15, 0.84) | 0.46 (0.16, 1.30) |

^1^Adjusted for maternal age, maternal education, parity, smoking status, diabetes, BMI, race/ethnicity (black, white, Hispanic, others), sex and year of birth

ASD: Autism Spectrum Disorder

## eTable 10: Sensitivity Analysis: Association between 5-methoxytryptophol and ASD, ADHD and other DD using a stringent comparator group

|  |  |  | ASD | | | ADHD | | | Other DD | | |
| --- | --- | --- | --- | --- | --- | --- | --- | --- | --- | --- | --- |
| 5-MTX | Total n | ND n | n | Crude | Adjusted^1^ | n | Crude | Adjusted^1^ | n | Crude | Adjusted^1^ |
| Continuous | 874 | 204 | 87 | 0.57 (0.43, 0.75) | 0.63 (0.45, 0.89) | 269 | 0.86 (0.72, 1.03) | 0.84 (0.68, 1.04) | 314 | 1.17 (0.96, 1.42) | 1.22 (0.98, 1.51) |
| Quartiles |  |  |  |  |  |  |  |  |  |  |  |
| Q1 | 219 | 41 | 42 | Ref | Ref | 86 | Ref | Ref | 50 | Ref | Ref |
| Q2 | 218 | 64 | 13 | 0.20 (0.10, 0.41) | 0.20 (0.08, 0.51) | 59 | 0.44 (0.26, 0.73) | 0.42 (0.23, 0.77) | 82 | 1.05 (0.62, 1.78) | 1.17 (0.66, 2.07) |
| Q3 | 219 | 58 | 14 | 0.24 (0.11, 0.49) | 0.31 (0.12, 0.79) | 50 | 0.41 (0.24, 0.70) | 0.34 (0.18, 0.64) | 97 | 1.37 (0.81, 2.32) | 1.77 (0.99, 3.17) |
| Q4 | 218 | 41 | 18 | 0.43 (0.21, 0.86) | 0.49 (0.20, 1.21) | 74 | 0.86 (0.51, 1.47) | 0.86 (0.46, 1.63) | 85 | 1.70 (0.97, 2.96) | 1.92 (1.05, 3.50) |
| Quartiles |  |  |  |  |  |  |  |  |  |  |  |
| Q1 | 219 | 41 | 42 | Ref | Ref | 86 | Ref | Ref | 50 | Ref | Ref |
| Q2-Q4 | 655 | 163 | 45 | 0.26 (0.16, 0.46) | 0.31 (0.15, 0.63) | 183 | 0.54 (0.35, 0.82) | 0.50 (0.30, 0.83) | 264 | 1.33 (0.84, 2.10) | 1.56 (0.94, 2.57) |
| Tertiles |  |  |  |  |  |  |  |  |  |  |  |
| T1 | 293 | 62 | 50 | Ref | Ref | 101 | Ref | Ref | 80 | Ref | Ref |
| T2 | 290 | 82 | 12 | 0.18 (0.09, 0.37) | 0.21 (0.08, 0.50) | 79 | 0.59 (0.38, 0.92) | 0.53 (0.32, 0.90) | 117 | 1.11 (0.72, 1.71) | 1.26 (0.78, 2.03) |
| T3 | 291 | 60 | 25 | 0.52 (0.28, 0.94) | 0.70 (0.33, 1.50) | 89 | 0.91 (0.58, 1.44) | 0.90 (0.53, 1.54) | 117 | 1.51 (0.96, 2.38) | 1.68 (1.03, 2.75) |
| Quintiles |  |  |  |  |  |  |  |  |  |  |  |
| Q1 | 177 | 30 | 36 | Ref | Ref | 73 | Ref | Ref | 38 | Ref | Ref |
| Q2 | 173 | 44 | 15 | 0.28 (0.13, 0.61) | 0.26 (0.10, 0.68) | 47 | 0.44 (0.24, 0.79) | 0.54 (0.27, 1.07) | 67 | 1.20 (0.65, 2.22) | 1.61 (0.82, 3.14) |
| Q3 | 175 | 53 | 9 | 0.14 (0.06, 0.33) | 0.12 (0.04, 0.36) | 53 | 0.41 (0.23, 0.73) | 0.32 (0.16, 0.64) | 60 | 0.89 (0.48, 1.64) | 1.05 (0.54, 2.05) |
| Q4 | 175 | 45 | 12 | 0.22 (0.10, 0.49) | 0.36 (0.13, 1.00) | 39 | 0.36 (0.19, 0.65) | 0.35 (0.17, 0.72) | 79 | 1.39 (0.76, 2.53) | 2.11 (1.09, 4.11) |
| Q5 | 174 | 32 | 15 | 0.39 (0.18, 0.85) | 0.40 (0.15, 1.11) | 57 | 0.73 (0.40, 1.34) | 0.85 (0.42, 1.75) | 70 | 1.73 (0.91, 3.26) | 2.09 (1.04, 4.19) |

1. Adjusted for maternal age, maternal education, parity, smoking status, diabetes, BMI, race/ethnicity (black, white, Hispanic, others), sex and year of birth

ASD: Autism Spectrum Disorder; ADHD: Attention-deficit/hyperactivity disorder; DD: Developmental disabilities; 5-MTX: 5-methoxytryptophol

## eTable 11: Neurodevelopmental conditions by time of birth

| Outcome^1^ | Deliveries between 8 am – 8 pm | Deliveries between 8 pm – 8 am |
| --- | --- | --- |
| Neurotypical | 159 (53.9%) | 136 (46.1%) |
| ASD | 44 (55.0%) | 36 (45.0%) |
| ADHD | 120 (57.4%) | 89 (42.6%) |
| Other DD | 149 (51.9%) | 138 (48.1%) |

^1^ASD: Autism Spectrum Disorder; ADHD: Attention-deficit/hyperactivity disorder; DD: Developmental disabilities
